# Stimuli-sensitive nano-drug delivery with programmable size changes to enhance accumulation of therapeutic agents in tumors

**DOI:** 10.1080/10717544.2023.2186312

**Published:** 2023-03-09

**Authors:** Mohammad Souri, Mohammad Kiani Shahvandi, Mohsen Chiani, Farshad Moradi Kashkooli, Ali Farhangi, Mohammad Reza Mehrabi, Arman Rahmim, Van M. Savage, M. Soltani

**Affiliations:** a Department of NanoBiotechnology, Pasteur Institute of Iran, Tehran, Iran; b Department of Mechanical Engineering, K. N. Toosi University of Technology, Tehran, Iran; c Departments of Radiology and Physics, University of British Columbia, Vancouver, British Columbia, Canada; d Department of Integrative Oncology, BC Cancer Research Institute, Vancouver, British Columbia, Canada; e Department of Ecology and Evolutionary Biology, University of California Los Angeles, Los Angeles, California, USA; f Department of Computational Medicine, David Geffen School of Medicine, University of California Los Angeles, Los Angeles, California, USA; g Santa Fe Institute, Santa Fe, New Mexico, USA; h Department of Electrical and Computer Engineering, University of Waterloo, Waterloo, Canada; i Centre for Biotechnology and Bioengineering (CBB), University of Waterloo, Waterloo, Canada; j Advanced Bioengineering Initiative Center, Multidisciplinary International Complex, K. N. Toosi University of Technology, Tehran, Iran

**Keywords:** Drug delivery, cancer nanomedicine, tumor penetration, hyperthermia, multi-stage delivery system, focused ultrasound, temperature-sensitive nanoparticles, pH-responsive nanoparticles

## Abstract

Nano-based drug delivery systems hold significant promise for cancer therapies. Presently, the poor accumulation of drug-carrying nanoparticles in tumors has limited their success. In this study, based on a combination of the paradigms of intravascular and extravascular drug release, an efficient nanosized drug delivery system with programmable size changes is introduced. Drug-loaded smaller nanoparticles (secondary nanoparticles), which are loaded inside larger nanoparticles (primary nanoparticles), are released within the microvascular network due to temperature field resulting from focused ultrasound. This leads to the scale of the drug delivery system decreasing by 7.5 to 150 times. Subsequently, smaller nanoparticles enter the tissue at high transvascular rates and achieve higher accumulation, leading to higher penetration depths. In response to the acidic pH of tumor microenvironment (according to the distribution of oxygen), they begin to release the drug doxorubicin at very slow rates (i.e., sustained release). To predict the performance and distribution of therapeutic agents, a semi-realistic microvascular network is first generated based on a sprouting angiogenesis model and the transport of therapeutic agents is then investigated based on a developed multi-compartment model. The results show that reducing the size of the primary and secondary nanoparticles can lead to higher cell death rate. In addition, tumor growth can be inhibited for a longer time by enhancing the bioavailability of the drug in the extracellular space. The proposed drug delivery system can be very promising in clinical applications. Furthermore, the proposed mathematical model is applicable to broader applications to predict the performance of drug delivery systems.

## 1. Introduction

Nanoparticles have been very promising in preclinical studies as a tool in drug delivery applications in cancer treatment (van der Meel et al., 2019; Jadidi et al., 2020; Poon et al., 2020; Kashkooli, Soltani, Souri et al., 2021; Soltani, Moradi Kashkooli et al., 2021). The high loading capacity of therapeutic agents is one of the most important properties of nanoparticles, which prevents their degradation by the biological environment (Mitchell et al., 2021; Jadidi et al., 2022; Souri, Soltani, Kashkooli et al., 2022). Meanwhile, stimulus-responsive nanoparticles drastically reduce the side effects of chemotherapy drugs by preventing their unwanted release (Karimi et al., 2016; Kashkooli et al., 2020; Soltani, Souri et al., 2021). After reaching the target site and accumulating in the tissue in response to an internal or external stimulus, the nanoparticles begin to release the drug slowly (sustainable release) or rapidly (burst release) (Karimi et al., 2016; Kashkooli et al., 2020; Namakshenas & Mojra, 2023). However, to accumulate nanoparticles in the tissue, they face various barriers (such as clearance and low transvascular rates) (Blanco et al., 2015). Nanoparticles are accumulated in tissue based on the enhanced permeability and retention (EPR) effect, known as passive targeting (Bertrand et al., 2014; Kashkooli et al., 2020; Kashkooli, Rezaeian et al., 2022; Abazari et al., 2023).

Recently, transport through transcellular channels or via endocytosis/transcytosis has been recognized as the dominant mechanism for nanoparticle extravasation (Sindhwani et al., 2020). However, various studies, regardless of the specific transvascular mechanism, have shown that the accumulation of particles in the tissue is very poor (Dai et al., 2018; Price et al., 2020). The physicochemical properties of nanoparticles play an important role in their transport and accumulation in tissues. Among them, nanoparticle size is a key parameter in nanoparticle transport (Ding et al., 2019; Souri, Soltani, Kashkooli et al., 2022). Nanoparticles with smaller sizes (less than 10 nm) compared to vascular pores and extracellular matrix pores have higher accumulation and penetration depth than their larger counterparts (Jain & Stylianopoulos, 2010; Souri, Soltani, Kashkooli, Shahvandi, 2022). However, small nanoparticles have a shorter circulation time due to the rapid clearance by the kidneys. They can also accumulate in normal tissues, leading to a variety of side effects (Yu & Zheng, 2015). By contrast, larger nanoparticles, although having a longer circulation time, exhibit less accumulation and penetration depths (Duan & Li, 2013). According to this, conventional nano-based drug delivery systems have not had significant success in inhibiting tumor growth and tumor eradication.

To overcome the problems of the bioavailability of free drugs in the extracellular space, the intravascular release paradigm has been developed (Zhan et al., 2019; Souri et al., 2021; Ten Hagen et al., 2021). In this paradigm, nanoparticles are considered to be larger because they do not need to accumulate in the tissue, have a longer circulation, and can carry more cargo. An important feature of the intravascular release paradigm is that it provides a very high local concentration of the drug in a relatively small volume. The intravascular release paradigm must include an important principle: the release of therapeutic agents at very high rates in response to stimuli. To achieve the highest therapeutic potential, rapid release and a high transvascular rate must overcome the high velocity of the bloodstream (Seynhaeve et al., 2020; Souri, Moradi Kashkooli et al., 2022). A great stimulus for using the intravascular paradigm is the thermal field because the response to the thermal field in plasma is faster (Seynhaeve et al., 2020; Souri, Moradi Kashkooli et al., 2022).

The intravascular release paradigm, although providing higher concentrations and higher penetrations, does not inhibit tumor growth for a long time due to the rapid uptake of the drug by cancer cells and the shorter life-time of the drug in the extracellular space (Souri et al., 2021). High penetration depth with a long half-life in extracellular space is a feature of a successful drug delivery system for tumor eradication. Multi-stage systems with the ability to change the size have so far shown remarkable success in various studies on inhibiting tumor growth (Wong et al., 2011; Hu et al., 2018; Xiong et al., 2020; Kashkooli, Soltani, Momeni et al., 2021). Wong et al. (2011) proposed a multi-stage system in which 100-nm gelatin nanoparticles release 10-nm quantum dots (QD) nanoparticles in response to matrix metalloproteinases (MMP-2) after their extravasation to the tumor microenvironment. The results demonstrated that the reduction in nanoparticle size led to improved diffusive transport efficacy. Dendrimers loaded with cisplatin encapsulated inside liposomal nanoplatforms are studied by Xiong et al. (2020). Due to nanoparticle size (162 nm), liposomes have a long blood circulation. After accumulation in the tissue, photothermal stimuli cause the release of tiny nanoparticles (8.6 nm) that are able to reach the deeper region. When there is less oxygen in a tumor, small size nanoparticles release the drug, which kills cancer cells. Hu et al. (2018) developed a delivery system with size changeable to improve tumor penetration. In this study, the small-sized dendrimeric prodrug of doxorubicin (DOX) (∼25 nm) was loaded in size shrinkable hyaluronic acid shells (∼200 nm). The larger nanoparticle was disintegrated in response to the enzymes present in the tumor tissue and released small nanoparticles. The smaller nanoparticles released their cargo after penetrating deeper regions in response to photothermal stimuli. Numerous studies have demonstrated that multi-stage drug delivery systems can circumvent biological barriers such a high clearance rate and a poor penetration depth. However, there is still a fundamental challenge: larger nanoparticles (greater than 100 nm) cannot considerably accumulate in the tissue, which may lead to an inadequate therapeutic response. Consequently, the purpose of this research is to acquire a substantial accumulation in addition to deepening the penetration.

**Figure 1.**
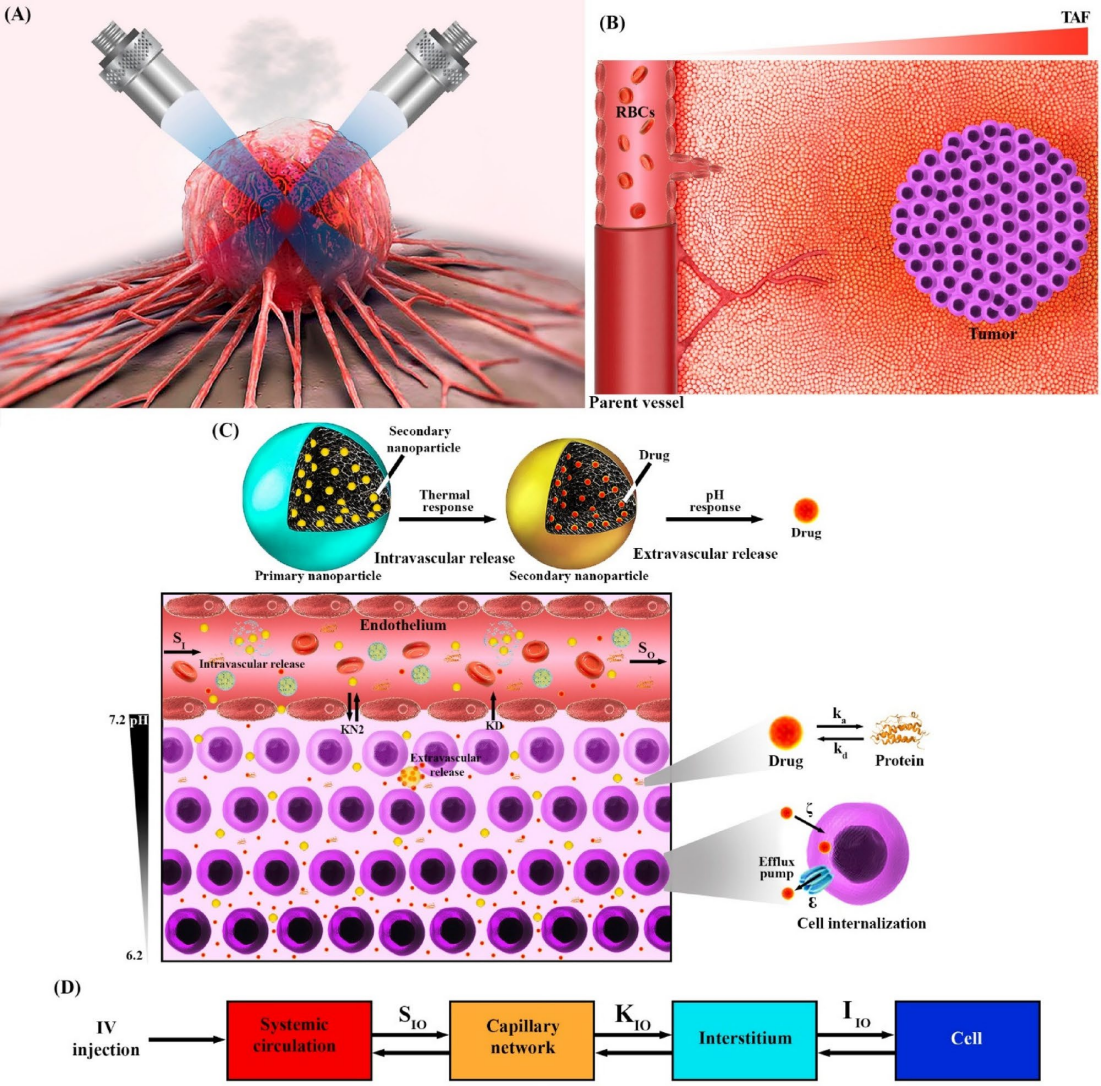
A graphic view of what is happening in the present study. A: Tumor tissue and blood in the microvasculature are heated under focused ultrasound by two convex transducers at a 90° cross angle [adapted from (Reviving Failed Antibody Treatments for Solid Tumors, Original story from UVA Cancer Center (April 7, 2021))]. B: New capillary sprouts from parent vessels based on tumor angiogenesis factors (TAFs) concentration form a new microvascular network. C: The increase in blood temperature causes the secondary nanoparticles to be released from the primary nanoparticles and enter the tissue across the endothelial barrier. Depending on the level of acidity and the distribution of oxygen (distance from the microvasculature), the secondary nanoparticles begin to release the drug at a certain rate. The drug in the extracellular space binds to proteins in the plasma, and the other part of it, which is located near the microvasculature, enters the bloodstream. Eventually, the remaining free drug can enter the cell and, if not excreted, can cause cell death by damaging the cell organelle. D: A schematic of the multi-compartment model presented in the present study. S: Exchange of therapeutic agents between systemic circulation and capillary network, K: Exchange of therapeutic agents between capillary network and interstitium, I: Exchange of therapeutic agents between interstitium and cell, $k_{a}$: Association with protein, $k_{d}$: Dissociation with protein, ε: Cellular efflux functions, ξ: Cellular uptake functions.

As such, in the present study, based on a comprehensive mathematical model, by combining the paradigms of intravascular and extravascular release, a new drug delivery system is presented to overcome problems such as poor tumor accumulation, poor tumor penetration, and poor bioavailability. In this way, smaller nanoparticles (secondary nanoparticles) that carry the drug are held in larger nanoparticles (primary nanoparticles), known as the ‘wrapping strategy’, and in response to a thermal field in the microvascular network of the tumor, are released. In fact, the primary nanoparticles disintegrate in response to temperature. Secondary nanoparticles, because of their small size, have a high transvascular rate and extravasate into the tissue in large amounts. The microvascular network is also generated based on a sprouting angiogenesis model (Abazari et al., 2022; Kashkooli, Abazari et al., 2022; Kiani Shahvandi et al., 2022). The thermal field is generated by ultrasonic power deposition in the blood and tissue. Here, two transducers with a 90° cross-angle are used, which provide the ability to create a wider thermal zone by creating several focal points. A controller is used to maintain the maximum temperature in the range of mild hyperthermia (less than 43 °C). After extravasation of secondary nanoparticles, in response to the tumor’s acidic environment, which depends on the distribution of oxygen, the drug begins to release at very low rates to kill cancer cells and inhibit tumor growth for a long time. Here, primary nanoparticles of diameters 150, 750, and 1040 nm are thought to transport higher amounts of drugs based on prior research (Chauhan et al., 2012; Igarashi et al., 2021). The selection of these sizes is intended to answer the question of whether or not an increase in the size of primary nanoparticles enhances accumulation in the tissue. Primary nanoparticles can be based on temperature-sensitive hydrogels or lysolipid-containing temperature-sensitive liposomes for burst release (Needham & Dewhirst, 2001; Zhao et al., 2016). Sizes of 1, 5, 12, and 20 nm are studied for secondary nanoparticles, all of which have a high diffusion coefficient and a high transvascular rate. Most pH-responsive nanoparticles are larger than 10 nm; nevertheless, in this study, we explore the role of ultrasmall nanoparticles (1 and 5 nm) as a further exploration of effect of size, even if they may not be practical in laboratory settings. Polycarbonate-based, mesoporous silica nanoparticles-based, polyacrylic acid functionalized $Co_{0.85}Se$ nanoparticles, ultra-small lipid–polymer hybrid nanoparticles, and ultrasmall gold nanoparticles are just some examples of the types of secondary nanoparticles that can respond to changes in pH (Wu et al., 2014; Dehaini et al., 2016; Yang et al., 2017; Ma et al., 2018; Wang et al., 2020). ***Figure 1*** gives an overview of what is happening in the present study.

## 2. Methods

A complete description of equations, parameters, and their values is provided in the Supplementary File. In the following, the physics involved and the governing equations are outlined.

**Figure 2.**
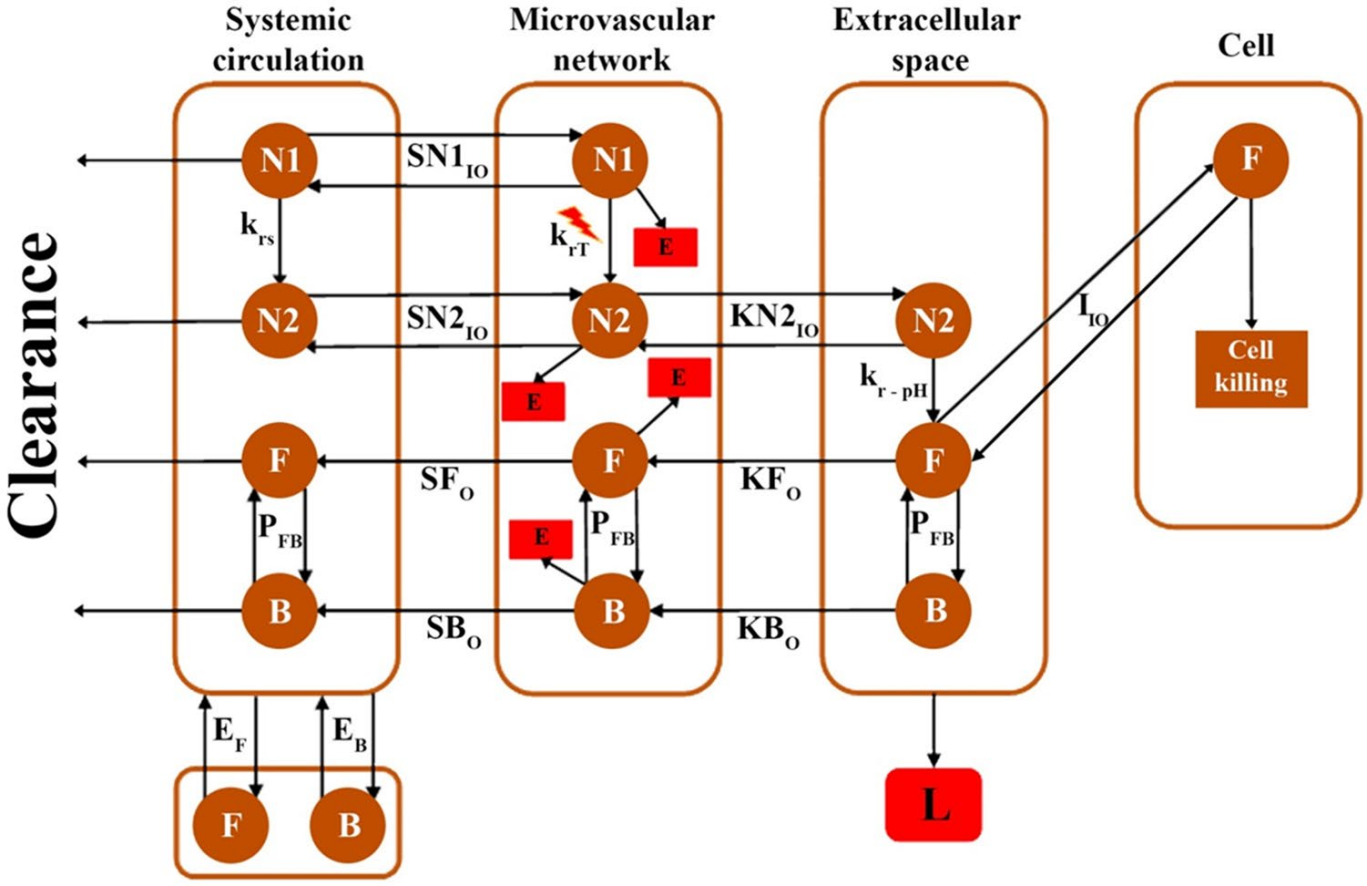
Multi-compartment model. Therapeutic agents are exchanged based on concentration gradients between compartments. Primary nanoparticles are not considered to enter the tissue due to their large size. Only the escape of free and bound drugs into the bloodstream is considered due to their low concentration. N1: Primary nanoparticles, N2: Secondary nanoparticles, F: Free drug, B: Bound drug, L: Lymphatic system, E: Elimination, $k_{\mathrm{rs}}$: Release rate at body temperature, $k_{\mathrm{rT}}$: Release rate at melting point, $k_{r-\mathrm{pH}}$: Release rate at acidic environment, $P_{\mathrm{FB}}$: Association/dissociation with protein, $E_{F}$: Free dox exchange with other body tissue, $E_{B}$: Bound dox exchange with other body tissue, ${SN1}_{\mathrm{IO}}$: Primary nanoparticle exchange between systemic circulation and microvascular network, ${SN2}_{\mathrm{IO}}$: Secondary nanoparticle exchange between systemic circulation and microvascular network, ${SN2}_{\mathrm{IO}}$: Secondary nanoparticle exchange between microvascular network and interstitium, ${\mathrm{SF}}_{O}$: Escape of free drug to the systemic circulation, ${\mathrm{SB}}_{O}$: Escape of bound drug to the systemic circulation, ${\mathrm{KF}}_{O}$: Escape of free drug to microvascular network, ${\mathrm{KB}}_{O}$: Escape of free drug to microvascular network, $I_{\mathrm{IO}}$: Free drug exchange between extracellular and intracellular space.

- *Angiogenesis*: The mathematical model predicts microvascular creation by following the movement of endothelial cells (ECs) at the microvascular sprout’s tip, which eventually becomes a microvascular network. In summary, this model considers three primary mechanisms for EC motion: random motility, chemotaxis, and haptotaxis (Anderson & Chaplain, 1998; Anderson et al., 2012).
(1) $$\frac{\partial n}{\partial t}=\underset{\text{random motility}}{\underbrace{D_{n}\nabla^{2}n}}-\underset{\text{chemotaxis and haptotaxis}}{\underbrace{\nabla \cdot [\chi (1+\delta c_{\mathrm{TAF}})n\nabla c_{\mathrm{TAF}}+\phi n\nabla f]}}$$
- *Hemodynamics and interstitial fluid flow*: The flow is divided into three sections for the fluid dynamics simulation model: intravascular blood flow, transvascular fluid flow, and interstitial fluid flow. The flow rate in each vessel is computed by using mass conservation at each network junction to investigate the incompressible flow through the microvascular network. As an exact solution to the Navier–Stokes fluid dynamics equation, Hagen–Poissville’s law can be applied to intravascular blood flow [Eq. (2)]. Starling’s law, which describes the interplay of oncotic and hydrostatic pressures in fluid movement through capillary membranes, is used to compute the transvascular fluid flow rate [Eq. (3)]. Interstitial fluid flow for the tissue is calculated by solving the governing equation for fluid flow through a porous medium (Darcy’s law) and adding source and sink terms for biological tissues [Eq. (4)] (Soltani & Chen, 2013; Kashkooli et al., 2019).
(2) $$Q_{b}=\frac{\pi }{128}\frac{\Delta P_{b}D^{4}}{L\;\mu (D,H)}$$
(3) $$Q_{\text{t}}=\pi DLL_{\text{p}}({\bar{P}}_{\text{b}}-\bar{P})$$
(4) $$\nabla \cdot v_{i}=\underset{\text{Source term }(\mathrm{vessels})}{\underbrace{\phi_{b}}}-\underset{\text{Sink term }(\text{lymph vessels}).}{\underbrace{\phi_{L}}}$$
- *Bioheat transfer and focused ultrasound*: In localized heating in tumor therapy, either blood vessels or tissue are heated. The temperature of tissue and blood can be calculated by solving the energy balance equations [Eq. (5)]. The heat is produced by an external stimulus which is generated by ultrasonic power deposition. The propagation of ultrasound in the medium is investigated by solving linear propagation of the pressure wave and is given by the Helmholtz equation [Eq. (6)]. To maintain the target temperature range during the simulation, a PI controller is used to adjust the temperature in the heating region [Eq. (7)] (Rezaeian et al., 2019; Namakshenas & Mojra, 2020).
(5) $$\rho c\frac{\partial T}{\partial t}={k\nabla }^{2}T-\underset{\text{The heat sink term }}{\underbrace{Q_{o}}}+\underset{\text{ The heat source term}}{K\cdot \underbrace{Q_{i}}}$$
(6) $$\frac{\kappa^{2}}{\rho }p+\nabla \cdot [\frac{1}{\rho }(\nabla p)]=0$$
  (7) $$K=K_{P}[T_{\mathrm{set}}-T(t)]+K_{i}\int [T_{\mathrm{set}}-T(t)]dt$$
- *Therapeutic agent distribution*: the transports and concentration distribution of therapeutic agents is presented based on a multi-compartment model (***Figure 2***). The distribution of therapeutic agents in circulation is based on clearance and mass exchange with the microvascular network. The extravasation of therapeutic agents from the vessels to the tissue is also defined by the pore model, which includes two terms convection and diffusion [Eq. (8)]. The distribution of therapeutic agents in the extracellular space is also presented based on the convection-diffusion-reaction (CDR) equation [Eq. (9)] (Soltani, 2013; Soltani & Chen, 2013).
(8) $$J_{\mathrm{transvascular}}=\underset{\text{Diffusion term}}{\underbrace{PA(C_{v}-C_{i})}}+\underset{\text{Convection term}}{\underbrace{L_{P}[P_{v}-P_{i}](1-\sigma )C_{v}}}$$
(9) $$\underset{\text{Interstitial concentration}}{\underbrace{\frac{\partial C_{i}}{\partial t}}}=\underset{\text{Diffusion term}}{\underbrace{D_{\mathrm{eff}}\nabla^{2}C_{i}}}-\underset{\text{Convection term}}{\underbrace{v\nabla C_{i}}}+\underset{\text{Source term}}{\underbrace{\varphi }}$$
- *Cell survival rate*: The change of tumor cell density with time is described by a pharmacodynamics model. This model is based on the intracellular concentration defined by three terms: the anticancer effect, physiological degradation, and cell proliferation [Eq. (10)] (Zhan et al., 2019).
(10) $$\underset{\text{Cell density}}{\underbrace{\frac{dD_{c}}{dt}}}=-\underset{\text{Anticancer effect}}{\underbrace{\frac{f_{\max}C_{i}}{{\mathrm{EC}}_{50}+C_{i}}D_{c}}}+\underset{\text{Cell proliferation}}{\underbrace{k_{c}D_{c}}}-\underset{\text{Physiological degradation}}{\underbrace{k_{g}D_{c}^{2}}}$$

## 3. Results

**Figure 3.**
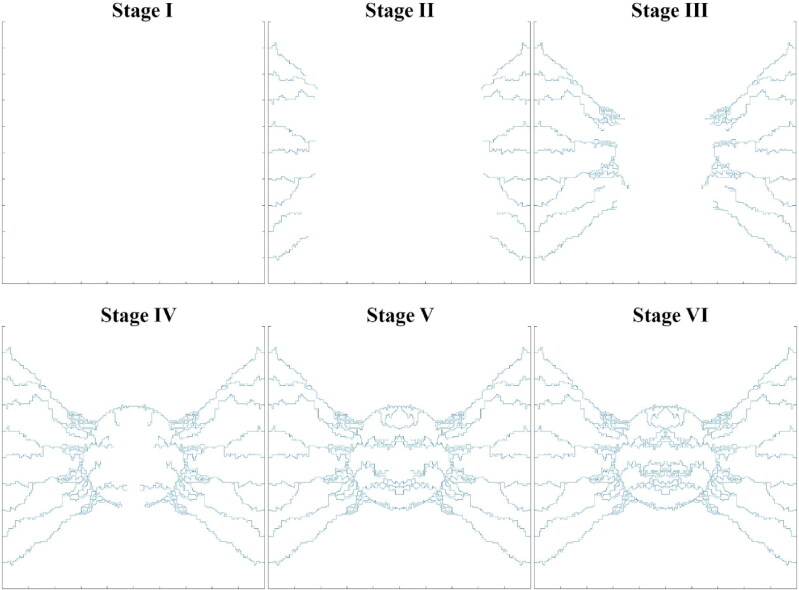
Different stages of microvascular network formation during the angiogenesis process.

The semi-realistic microvascular network is based on a hybrid continuous-discrete sprouting angiogenesis method that has different densities based on tumor angiogenesis factors (TAFs) concentration in different regions. In general, the angiogenesis process is such that the TAFs secreted by the oxygen-deficient cancerous cells reach the parent vessels and cause new capillary sprouts to form new vessels. Then the generated vessels are elongated, branched, deformed, and spread and converge toward the tumor where the concentration of TAFs is high. Eventually, new vessels expand inside the tumor and form microvascular loops (***Figure 3***).

**Figure 4.**
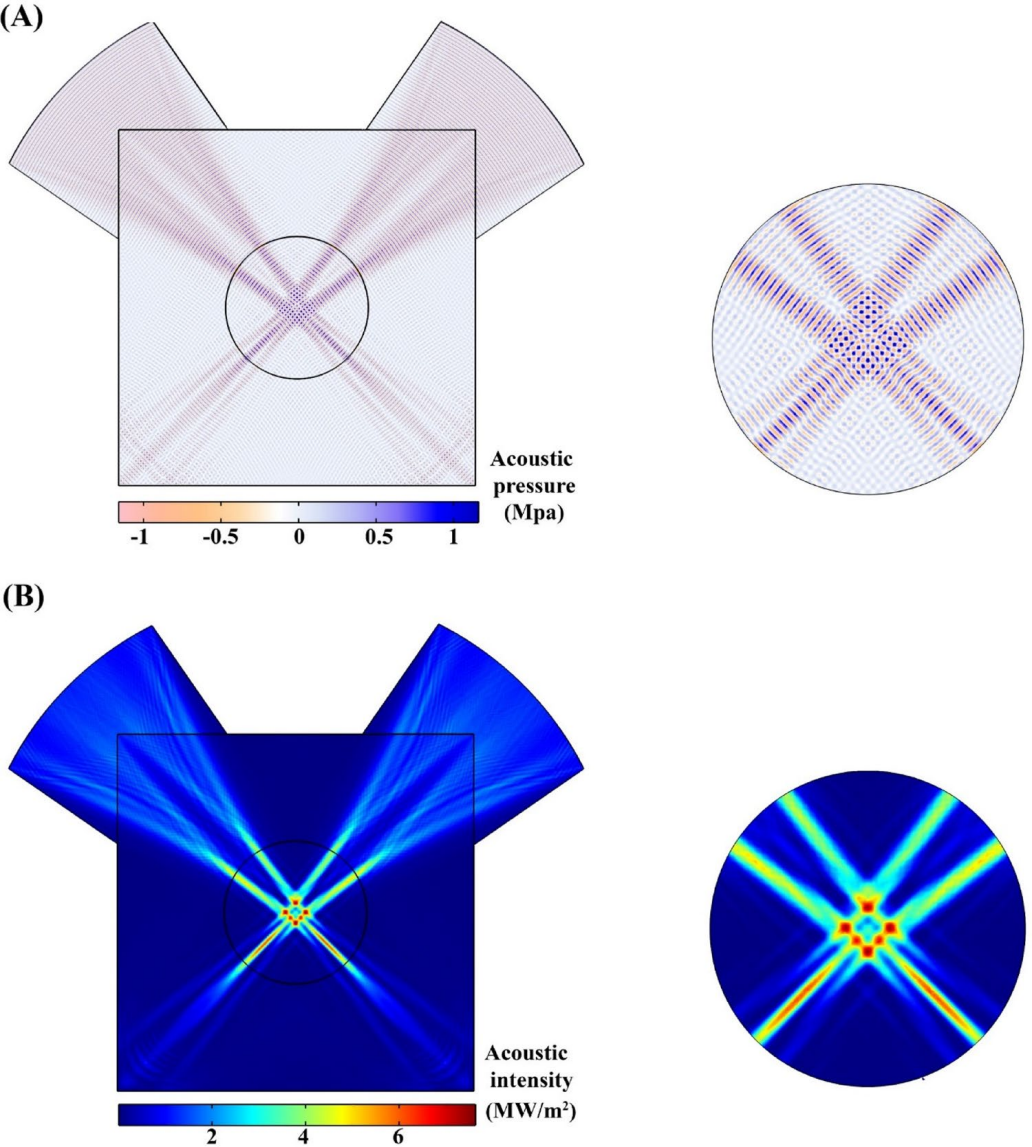
Distribution of pressure and acoustic intensity fields produced by focused ultrasound; The focus area in the center of the tumor has the highest pressure and acoustic intensity. The use of two transducers causes several focal points to be generated.

**Figure 5.**
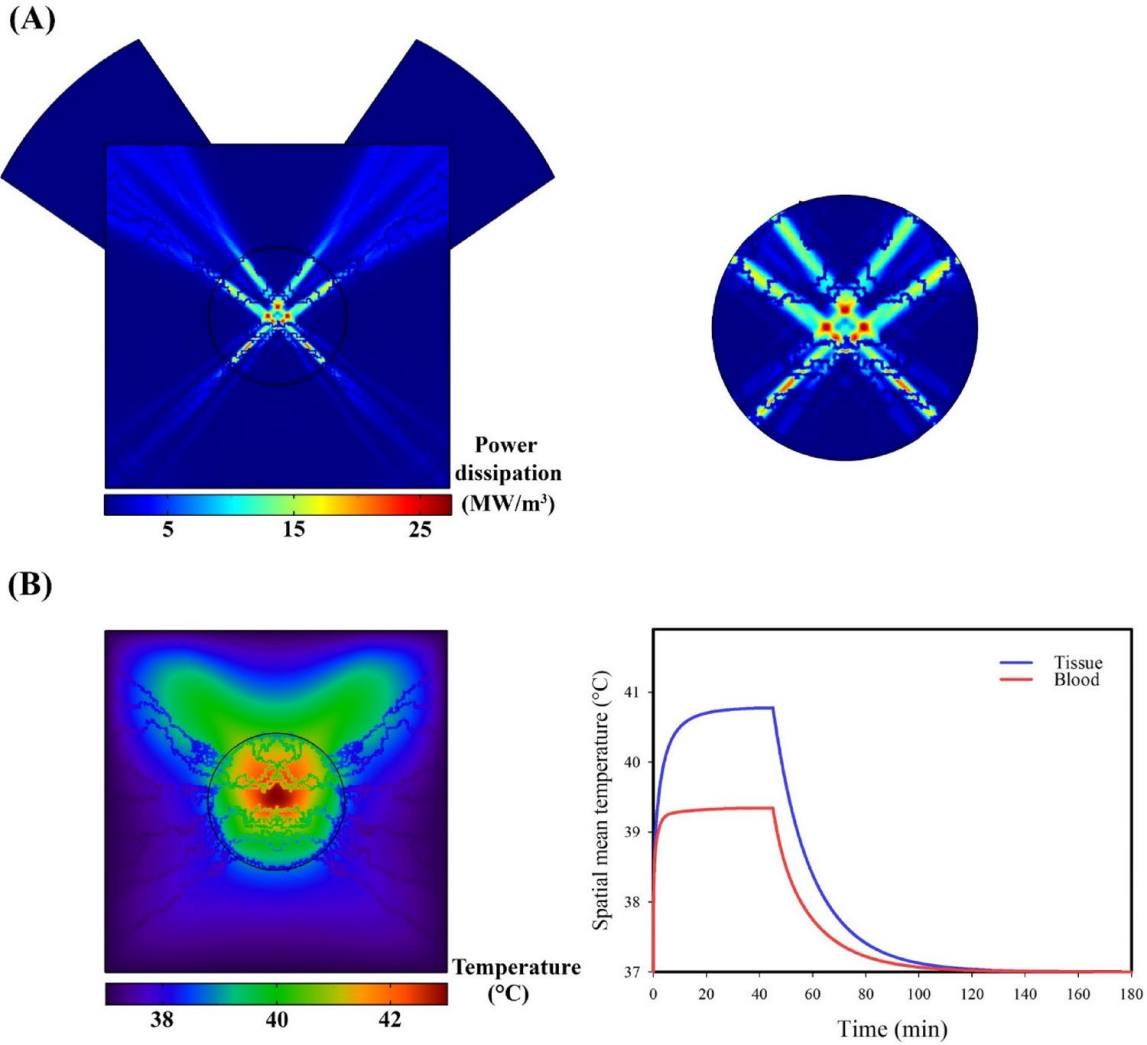
The ultrasonic power deposition by the focal points causes the central region of the tissue to be exposed to high temperatures. After exposure of ultrasound, the tissue temperature rises rapidly. Then, tissue temperature is kept in the mild hyperthermia range by the controller for 45 minutes.

To increase the temperature of tissue and blood, as mentioned before, two transducers with the same power (30 W) and frequency (2 MHz) are considered. According to a previous study that reported the optimal cross-angle of two transducers to be 90° (Kim et al., 2021), in this study, the transducers were embedded in such a way that their cross-angles were perpendicular to each other in the center of the tumor. The highest acoustic pressure and, consequently, the highest acoustic intensity were recorded in the focus area (***Figure 4***). The ultrasonic power deposition by the blood and tissue increases the temperature. Focused ultrasound with its controller is applied to maintain the temperature in the hyperthermia range for 45 min. The temperature of the tissue and blood rises rapidly, and slowly decreases after the ultrasound is offed (***Figure 5***). Increasing the temperature causes the release of secondary nanoparticles in the microvascular network. Concentration transport is considered by a multi-compartment model in which therapeutic agents are exchanged between compartments (Figure 2). In the following, the effects of secondary and primary nanoparticle size are evaluated as a key parameter in the performance of the present size-changeable drug delivery system.

### 3.1. Effect of secondary nanoparticles size

**Figure 6.**
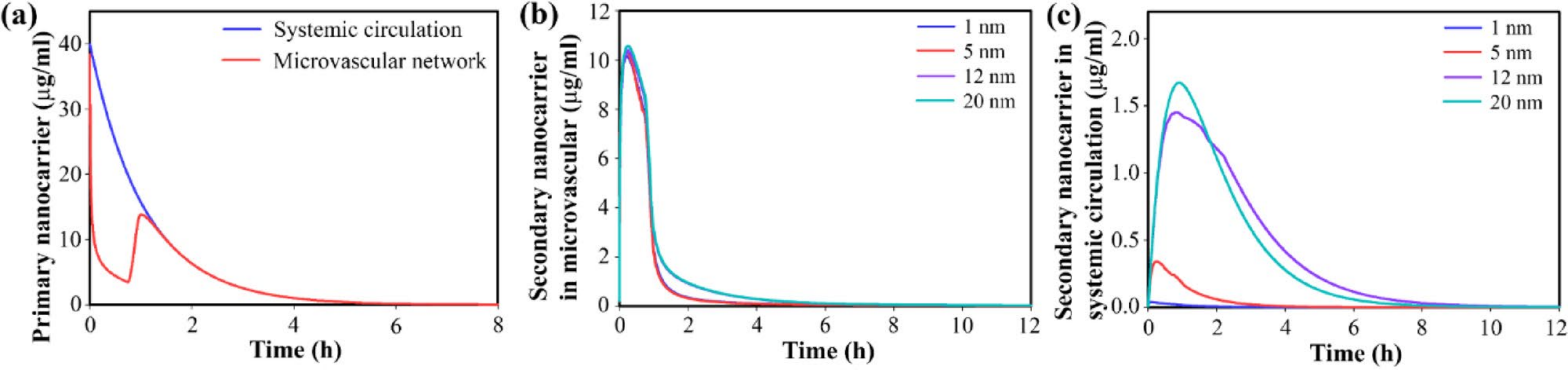
Temporal distribution of nanocarriers in blood stream by considering secondary nanoparticles in different sizes, for 150-nm primary nanoparticles; Temporal distribution of (a) primary nanoparticles in circulation and tumor microvascular network, (b) secondary nanoparticles in tumor microvascular network, (c) secondary nanoparticles in circulation.

First, 150 nm primary nanoparticles are considered, which carry secondary nanoparticles in sizes of 1, 5, 12, and 20 nm. This means that the scale of the drug delivery system decreases from 7.5 to 150 times within the microvascular network. This will increase tumor accumulation and then increase the penetration depth. The primary nanoparticles are not small enough to be quickly excreted by the kidneys, nor are they large enough to be trapped by the spleen and liver. Meanwhile, they are large enough to carry a significant load. Furthermore, the size of the primary nanoparticles is larger than 0.6 of the tumor vessel pore size (200 nm), so they do not pass through the pores due to hydrodynamic and electrostatic interactions (Chauhan et al., 2012; Stylianopoulos et al., 2018). Primary nanoparticles in the microvascular network of the tumor release their cargo rapidly in response to temperature, so their concentration decreases sharply during the period of hyperthermia in the microvascular network. However, these changes do not affect the concentrations in the circulation because the volume of tumor plasma is very small compared to the total volume $\left(V_{\mathrm{Tp}}/V_{\mathrm{Sp}}\right)$ (***Figure 6***(a)).

In a rapid response to temperature, secondary nanoparticles are released within the tumor microvascular network and provide high concentrations (Figure 6(b)). The loading capacity is examined in terms of concentration, so the amount of loading for the primary and secondary nanoparticles in different sizes is considered equal (although they are very different in terms of the number of loaded particles). Hence, the concentration peak of secondary nanoparticles of various sizes in the microvascular network is equal. Due to the blood flow, some of the released secondary nanoparticles, which do not have the opportunity to enter the tissue, enter the systemic circulation. Smaller nanoparticles are removed rapidly due to their high clearance rate (Figure 6(c)).

**Figure 7.**
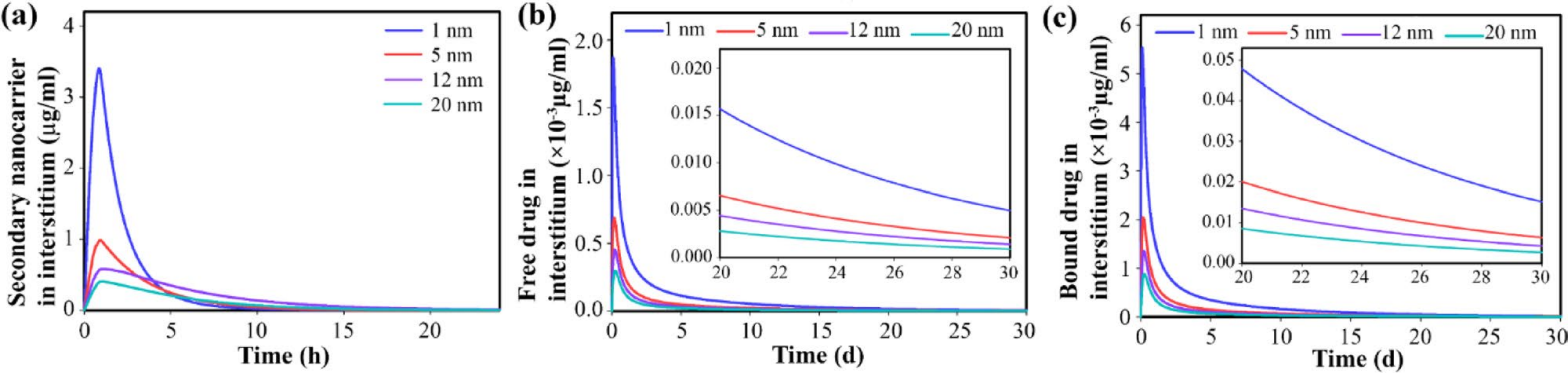
Temporal distribution of therapeutic agents inside the tumor interstitium by considering secondary nanoparticles in different sizes, for 150-nm primary nanoparticles; Temporal distribution of (a) secondary nanoparticles in extracellular space, (b) free drugs in extracellular space, (c) bound drugs in extracellular space. Smaller secondary nanoparticles exhibit improved therapeutic response by providing a higher concentration of free drugs in the interstitium.

Other amounts of the released secondary nanoparticles enter the extracellular space at certain rates due to their size. Smaller nanoparticles have a higher transvascular rate, so they accumulate at higher concentrations in the extracellular space (***Figure 7***(a)). Accumulated secondary nanoparticles in the extracellular space, in response to the acidic environment of the tumor, begin to release the drug at a very slow rate ∼10^−5 ^s^−1^. Hence, the free drug is available for longer durations. High concentrations of secondary nanoparticles also provide high concentrations of free drugs in the extracellular space (Figure 7(b)). Part of the free drug binds to proteins in the extracellular space (albumin) as a result of a dynamic process (association/dissociation). Hence, the concentration changes for the bound drug are similar to the trend of free drugs in the extracellular space (Figure 7(c)).

**Figure 8.**
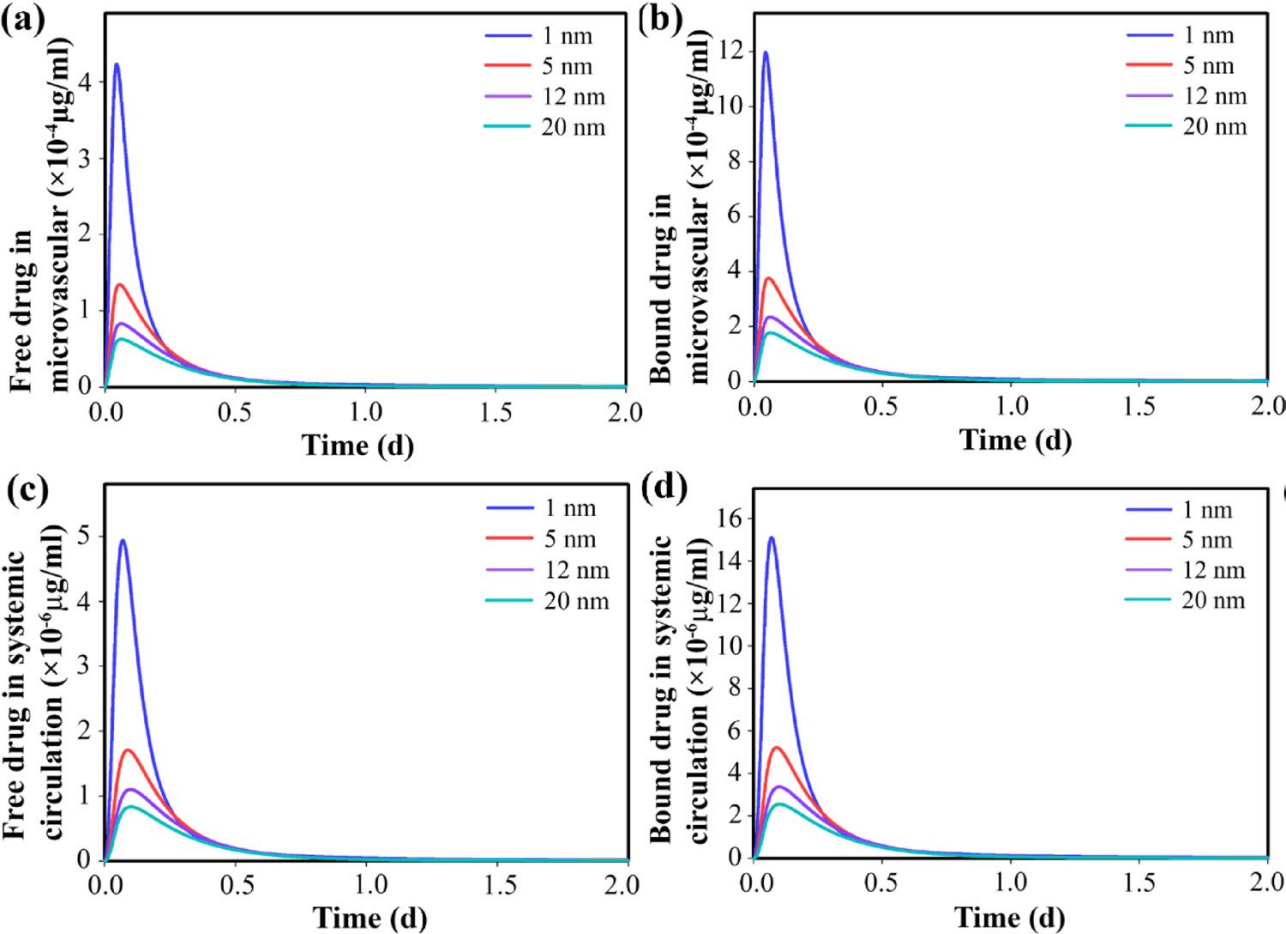
Temporal distribution of free- and bound drugs in blood stream by considering secondary nanoparticles in different sizes, for 150-nm primary nanoparticles; Temporal distribution of (a) free drugs in tumor microvascular network, (b) bound drugs in tumor microvascular network, (c) free drugs in circulation, (d) bound drugs in circulation.

**Figure 9.**
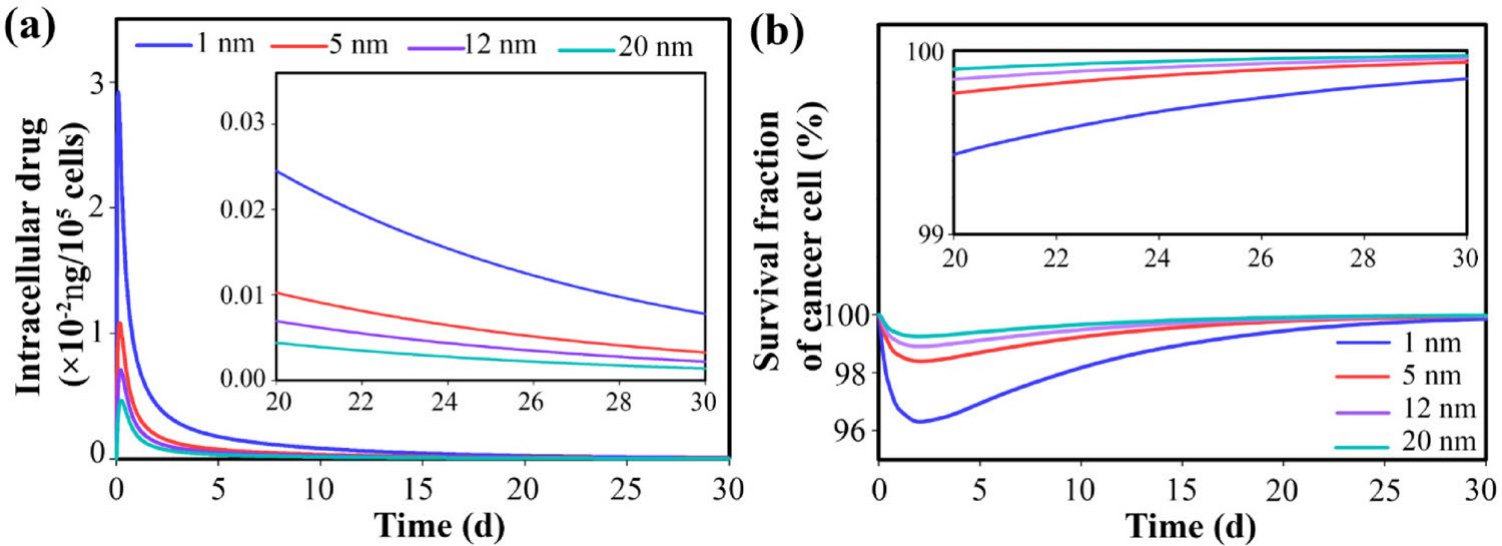
Temporal distribution of (a) internalized free drug and (b) survival fraction of tumor cells. Smaller secondary nanoparticles exhibit improved therapeutic response by providing a higher concentration of free drugs in the interstitium.

Free and bound drugs near the microvascular wall can enter the microvascular network and the systemic circulation through the bloodstream. During the circulation, the dynamic process of association/dissociation of the free drug with the protein continues (***Figure 8***(a–d)). The free drug in the extracellular space is exchanged with the intracellular space, so changes in the concentration of the drug inside the cell are the same as in the extracellular space through the cell membrane (***Figure 9***(a)). Slow release causes the cancer cells to be exposed to the free drug for a long time, thus inhibiting tumor growth (compared to the initial state) for a long time. The availability of drugs in higher concentrations also leads to higher cell death. However, due to the trend of concentration changes, after about 3 days, cell proliferation overcomes doxorubicin-induced cell death and cell density begins to increase (Figure 9(b)). Although the amount of drug remaining affects the cell proliferation rate, therefore, it is known that in the case of using secondary nanoparticles (size 1 nm), it takes 30 days for the cell density to reach close to the initial level, while in other cases it decreases to 15 days.

**Figure 10.**
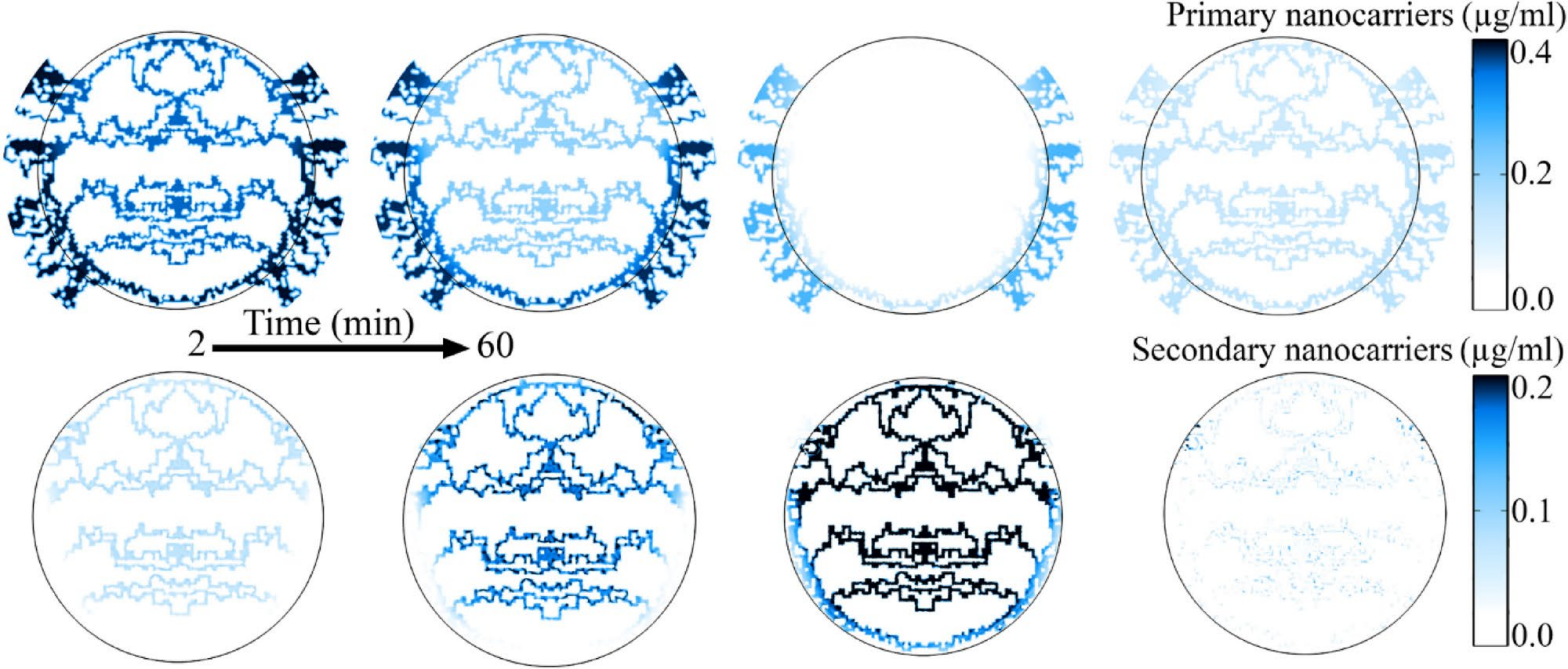
Spatial distribution of noncarriers. In the microvascular network at the time of hyperthermia, due to release operation, the primary nanoparticles have the lowest concentration while the secondary nanoparticles have the highest concentration.

**Figure 11.**
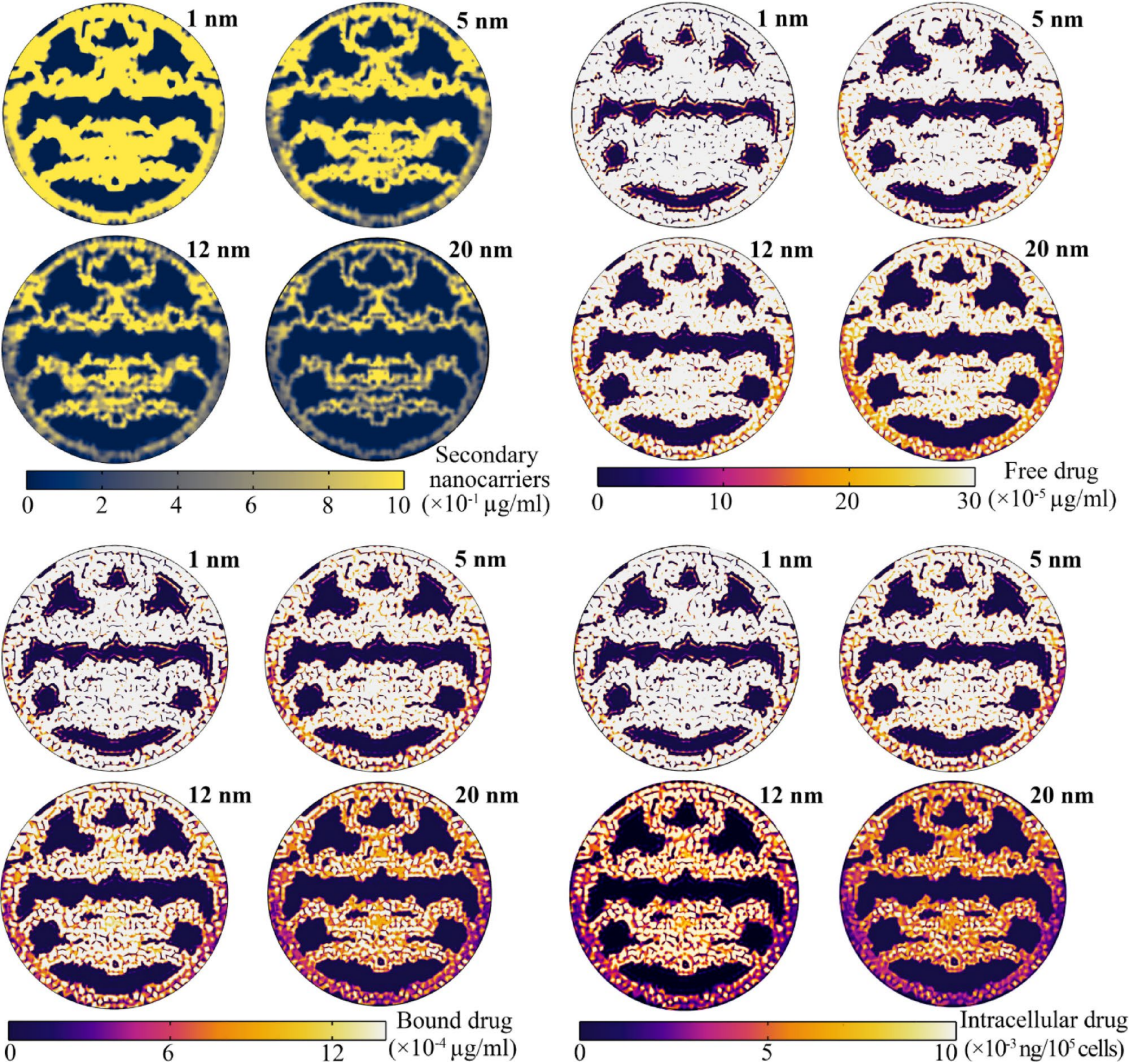
Spatial distribution of therapeutic agents inside the tumor interstitium at maximum value. Smaller secondary nanoparticles have higher concentrations and higher penetration depths than their larger counterparts. This causes the penetration depth and concentration of the free drug, followed by the internalized drug, to be greater.

In microvascular space that are heated to temperatures beyond the melting point of primary nanoparticles, the release rate is very high. Therefore, these regions have the highest concentration of secondary nanoparticles and the lowest primary nanoparticles within the microvascular network during the period of hyperthermia (***Figure 10***). After the heating exposure by focused ultrasound and the subsequent reduction in tissue temperature, the concentration of primary nanoparticles in the microvessel network increases, while the concentration of secondary nanoparticles decreases due to extravasation into the tumor tissue and exchange with the circulatory system. ***Figure 11*** shows the distribution of nanoparticles and free- and bound drugs in the extracellular and intracellular space. Smaller nanoparticles enter the extracellular space more easily from the microvascular network and at a higher concentration due to the diffusion mechanism. The spatial distribution of the secondary nanoparticles indicates that the reduction in size increases the accumulation of particles, which then increases the depth of penetration based on the diffusion mechanism. This has caused the free- and bound drug, followed by the internalized drug, to achieve a higher penetration depth in addition to higher concentration.

### 3.2. Effect of primary nanoparticles size

**Figure 12.**
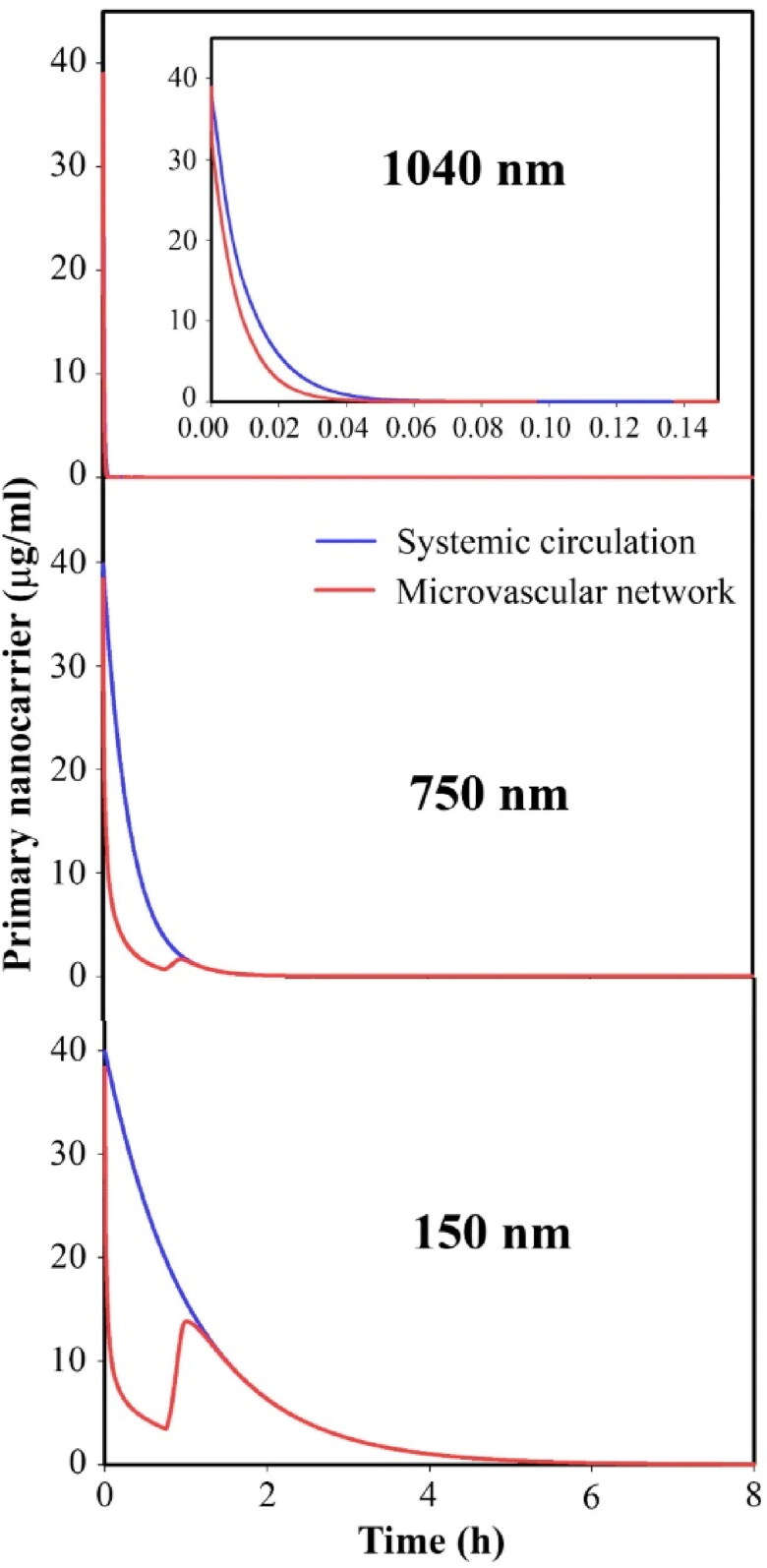
Temporal distribution of primary nanoparticles in circulation and tumor microvascular network.

**Figure 13.**
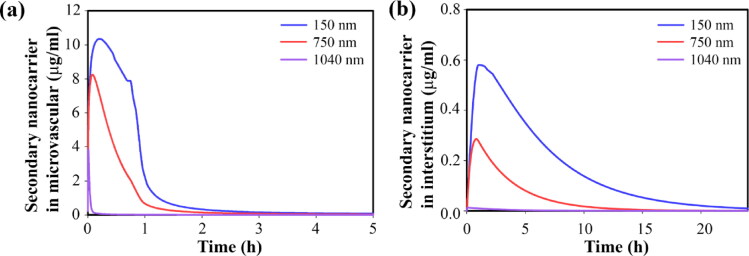
Temporal distribution of secondary nanoparticles of 12 nm (a) in tumor microvascular network, (b) in extracellular space.

**Figure 14.**
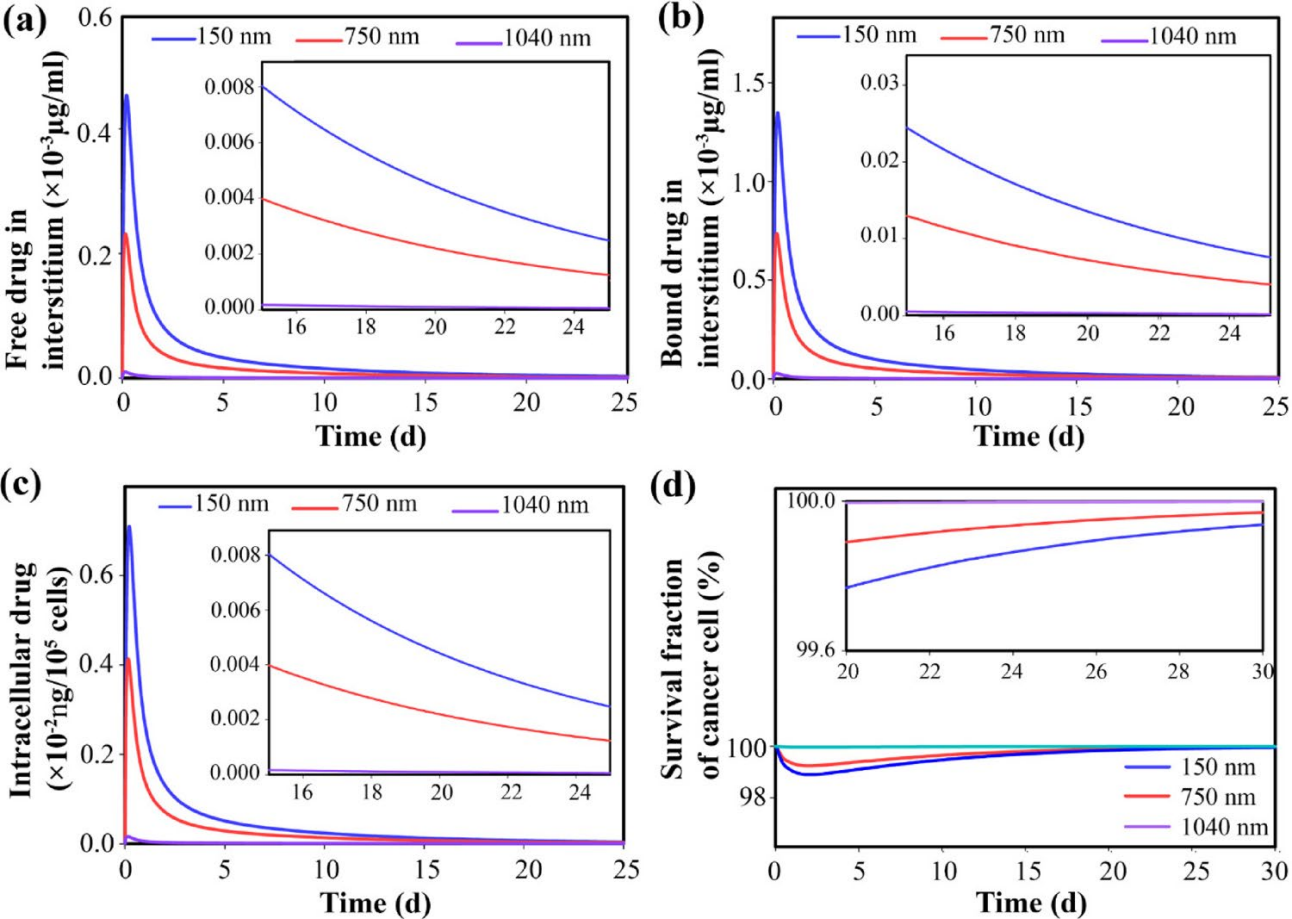
Temporal distribution of therapeutic agents and cell death considering 12 nm secondary nanoparticles and primary nanoparticles in different sizes, Temporal distribution of (a) free drugs in extracellular space, (b) bound drugs in extracellular space, (c) internalized free drug, and (d) survival fraction of tumor cells. Primary nanoparticles with smaller sizes have longer circulation time; hence, secondary nanoparticles accumulate in the tissue for a longer time and at a higher concentration. This improves bioavailability and therapeutic response.

Circulation time is one of the key parameters of primary nanoparticles that affects the performance of the current drug delivery system. In what follows, the effect of the size of primary nanoparticles that have different circulation times is investigated. As reported in other studies, larger nanoparticles are easily removed from circulation by the spleen. ***Figure 12*** shows the circulation times of primary particles in sizes of 150, 750, and 1040 nm. Briefly, 150 nm nanoparticles circulate for 5h, while 1040 nm nanoparticles circulate for only 5 min. Short-term circulation causes the concentration of primary nanoparticles to not be high enough (for a long time) to provide a high concentration of secondary nanoparticles in the microvascular network (***Figure 13***(a)). Therefore, high concentrations of secondary nanoparticles do not accumulate in the extracellular space (Figure 13(b)). As a result, a high concentration of the free drug, followed by the bound drug in the extracellular space, is not provided long-term (***Figure 14***(a,b)). Therefore, the drug amount that enters the cell is very small (Figure 14(c)). This reduces the drug’s toxicity on cell growth, so tumor growth is not inhibited. Figure 14(d) shows that the very low circulation time of primary nanoparticles of 1040 nm in size is not able to reduce cell density and inhibit tumor growth. Whereas the use of primary nanoparticles with long-term circulation has increased tumor cell death and inhibited tumor growth for a longer period.

## 4. Validation

**Figure 15.**
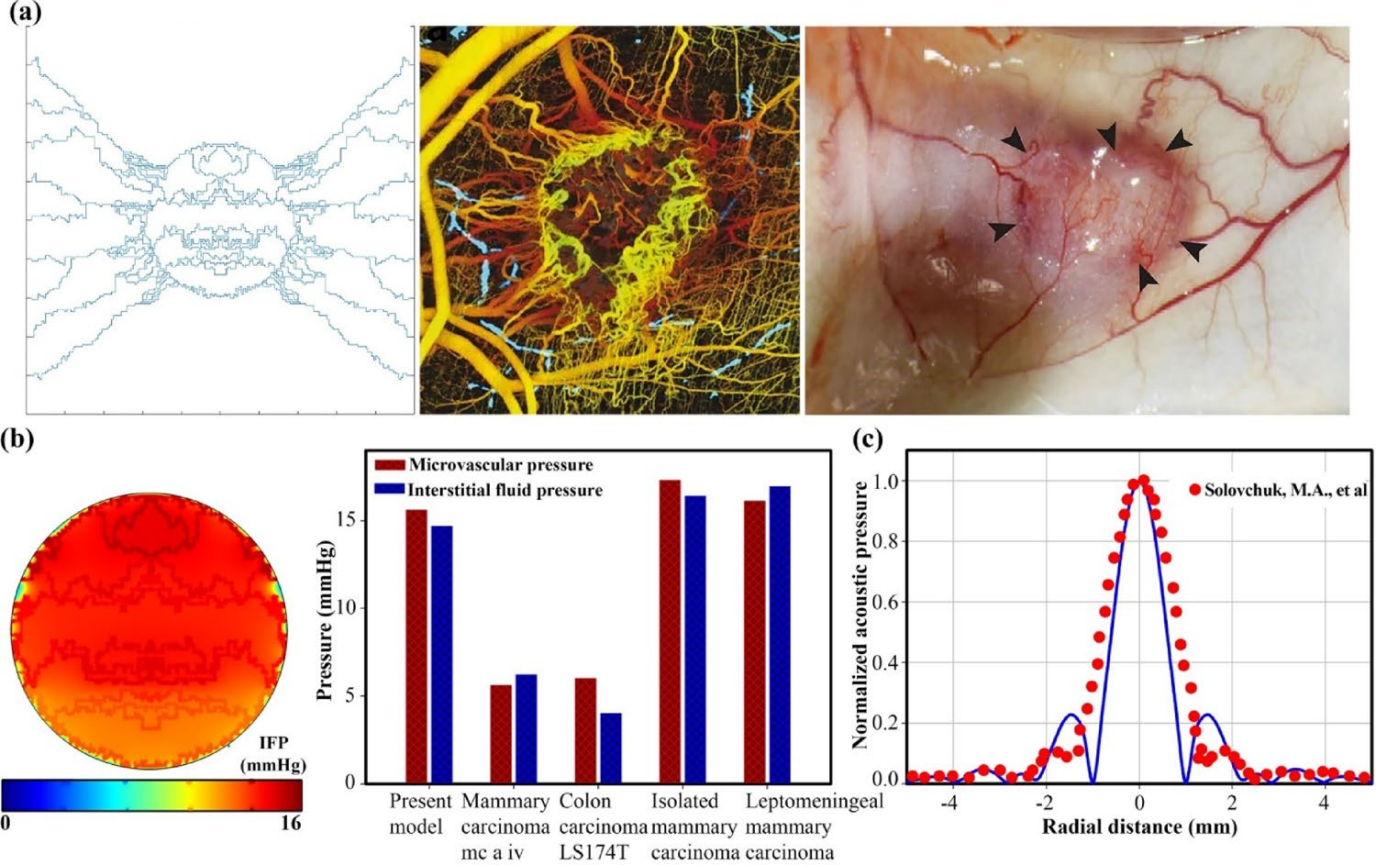
Validation of the present model; (a) A qualitative comparison of angiogenesis modeling in agreement with *in vivo* observations (Vakoc et al., 2009; Roudnicky et al., 2018) shows that the new vessels near the tumor border and inside the tumor have many branches and loops (Middle figure from (Vakoc et al., 2009), Right figure from (Roudnicky et al., 2018)). (b) A comparison of intravascular and interstitial pressure in agreement with different tumors in real models (Stylianopoulos et al., 2018) indicates their high pressure and low difference. (c) The spatial distribution of the normalized acoustic pressure for the single transducer of the present model in agreement with the experimental model (Solovchuk et al., 2012) shows that the maximum acoustic pressure is recorded at the focal point.

To ensure the validity of the present model, some of the results are qualitatively and quantitatively compared with experimental studies. Measuring concentration in *in vivo* models is very difficult if not impossible. On the other hand, the current drug delivery system is presented for the first time, so the results are not available to validate the concentration changes. Therefore, other factors that affect the results and concentration values are evaluated. A qualitative comparison of angiogenesis morphology of the present study with biological observations shows that what happens in the biological environment is also considered in the mathematical model, for example, formation of new capillary sprout, new microvascular branching near the tumor, and formation of microvascular loops. Also, within the tumor based on the concentration of TAFs varies microvascular density and microvascular loops (***Figure 15***(a)). The distribution of therapeutic agents is also affected by the flow of interstitial fluid (with less effect than diffusion). Hence, the interstitial fluid pressure (IFP) is also validated. The lack of an efficient lymphatic system in the tumor caused the IFP to be approximately equal to the intravascular pressure of the tumor. Tumor IFP has been reported to vary from 5.8 to 30 mmHg (Boucher & Jain, 1992; Soltani & Chen, 2011). Figure 15(b) shows that the estimated intravascular pressure and IFP in the present model are consistent with the reported experimental results. For acoustic validation, the normalized acoustic pressure when one transducer is used is compared with the experimental results. It is known that according to the experimental results, the highest pressure is recorded at the focal point and the acoustic pressure decreases with distance in the lateral direction (Figure 15(c)). In addition, the current model confirms the results of previous research (Yong et al., 2019; Gao et al., 2011; Xiong et al., 2020) on sustained release, demonstrating that it reduces tumor growth rate over a long period of time. The equations, assumptions, and parameters used in the present modeling are confirmed by other valid studies based on experimental results (Andriyanov et al., 2014; Mpekris et al., 2017; Igarashi et al., 2021; Mpekris et al., 2022). Indeed, mathematical models, including the one presented here, have been tested and examined in a wide range of research contexts for over 30 years (Anderson & Quaranta, 2008; Barbolosi et al., 2016). In light of these issues, the findings of the current model that presents a novel multi-stage drug delivery system can be trusted.

## 5. Discussion

Existing problems with nanoparticles such as low transvascular rates, poor accumulation in the extracellular space of tumors, and poor penetrations, led us to provide a multi-stage drug delivery system to solve the existing problems. The proposed drug delivery system has the potential to inhibit tumor growth for a long time owing to killing cancer cells by providing a high accumulation of active agent-carrying nanoparticles with sustained-release in addition to achieving high penetration depth. The temperature-responsive of the primary nanoparticles causes the secondary nanoparticles to be rapidly released only at high temperatures and to be released at very low rates at body temperatures (due to the unstable nature of the primary nanoparticles); hence, unwanted side effects of chemotherapy agents are prevented. Not only is the amount released at body temperature not high enough to counteract tumor growth, but the concentration of finer nanoparticles is not high enough to cause toxicity to other organs (Supplementary file, Figure S1). In regions where the temperature rises, smaller particles enter the tissue quickly, and over time, due to the slow-release rate in the extracellular space, their accumulation increases. After zero concentration of secondary particles in the microvascular network and systemic circulation, secondary nanoparticles in the extracellular space near the microvascular can enter the bloodstream. Meanwhile, 1-nm secondary nanoparticles enter the bloodstream at a higher rate, so their concentration in the tissue decreases faster (Supplementary Video 1). In general, smaller nanoparticles, although entering the tissue at a higher rate, also exit the tissue at a higher rate too (Supplementary file, Figure S2).

**Figure 16.**
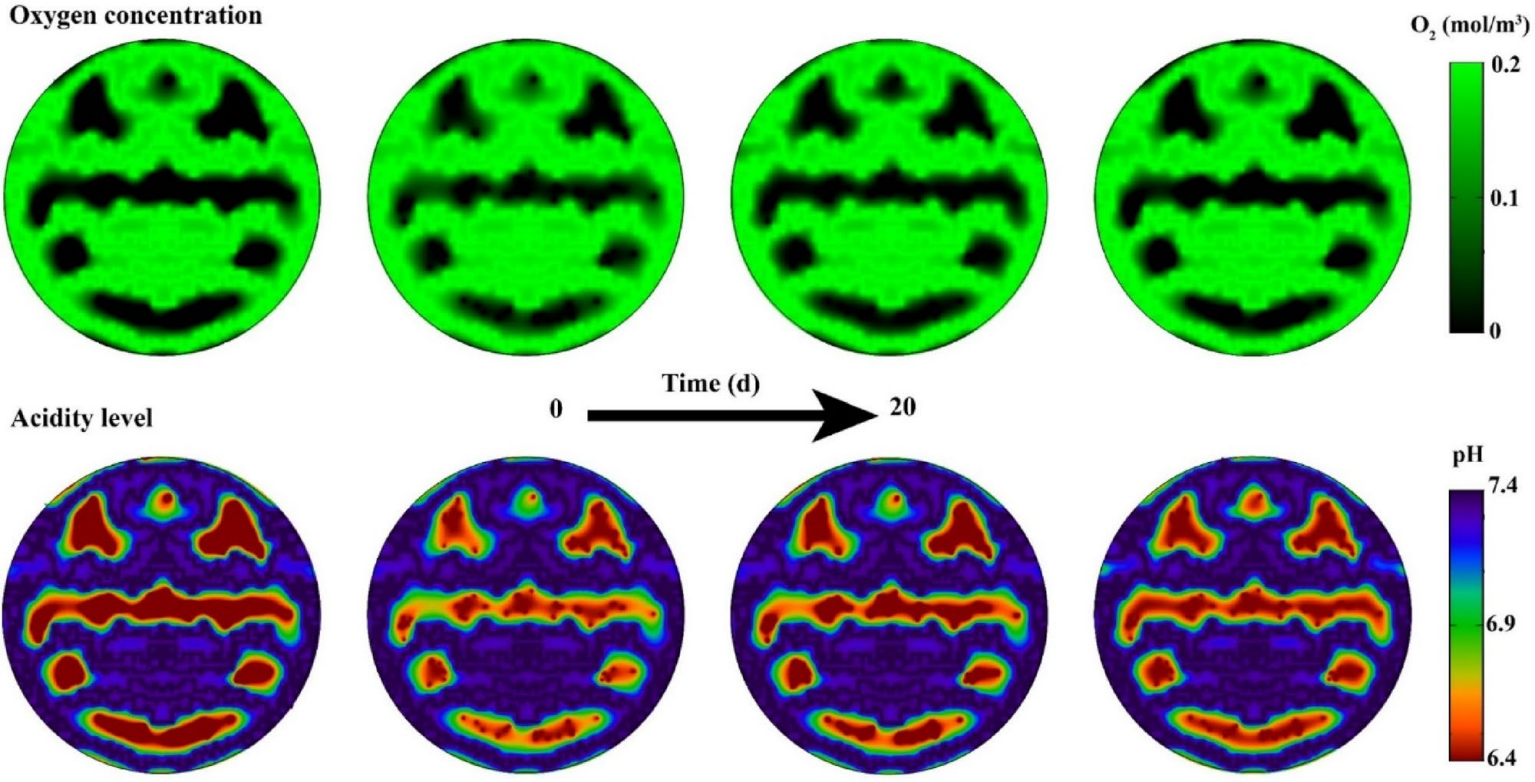
Oxygen distribution and acidity levels in tissues; There is a higher concentration of oxygen near the microvascular. This reduces lactic acid in these areas, so the areas near the microvascular have a higher pH level compared to longer distances from the microvascular. Decreasing cell density also increases oxygen concentration and decreases acidity levels.

**Figure 17.**
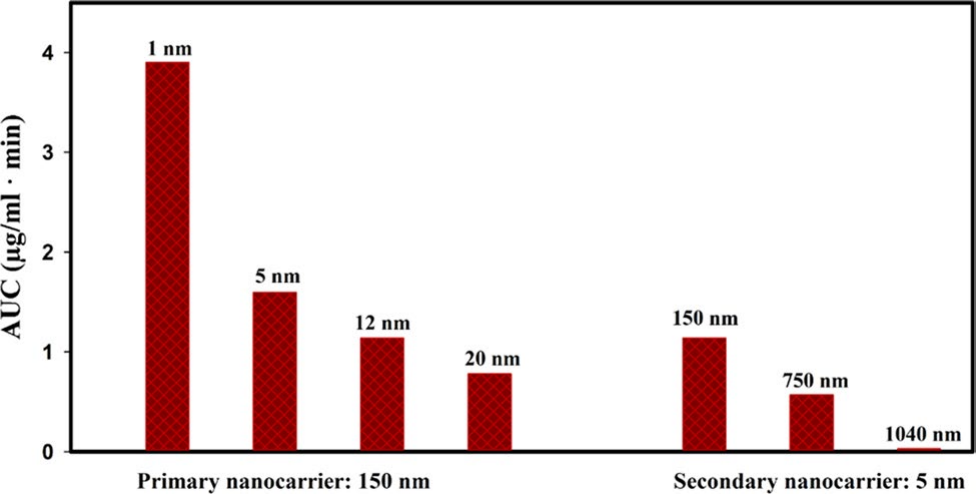
The area under curve (AUC) for the free drugs in extracellular space for different cases; The smaller secondary nanoparticles have higher accumulation in the tissue, which in turn provides a higher concentration of free drug for a longer time, thus improving the bioavailability of the free drug. On the other hand, increasing the circulation time by decreasing the size of the primary nanoparticles also increases the AUC.

Due to the distribution of oxygen in tumor tissue, which depends on the cell density, regions of the tumor that are further away from the microvascular have a higher level of acidity, so secondary nanoparticles in the extracellular space have different release rates depending on their location (***Figure 16***). The released drug can gain more penetration over time if not used by cancer cells (Supplementary Video 2). Another part of the free drug, which is located near the microvascular, enters the bloodstream. The drug released from the 1 nm secondary nanoparticles is expected to enter the bloodstream more due to its higher concentration in the tissue (Supplementary file, Figure S3). In general, the results show that reducing the size of secondary nanoparticles and increasing the circulation time by reducing the size of primary nanoparticles improves the bioavailability of free drugs or in other words increases the area under curve (AUC) of the free drug in the extracellular space (***Figure 17***). Improving bioavailability increases cell death. Increasing the injection concentration also increases the concentration of free drugs in the extracellular space. Therefore, cancer cells are exposed to higher concentrations of the drug, so cell death increases (Supplementary file, Figure S4), although injectable dose limits should be considered.

The proposed drug delivery system can enable effective therapies. At the same time, there are some concerns about this delivery system. Because some of the tiny nanoparticles enter the systemic circulation along with the bloodstream, they can accumulate in other organs and cause unwanted toxicity. For example, it has been reported that small particles can accumulate in the central nervous system and disrupt the body’s nervous system (Feng et al., 2015). Therefore, it is necessary to test this drug delivery system in different animal models and evaluate it for long periods after use to identify possible unwanted toxicities.

*In vivo* models can well estimate the efficiency of the current drug delivery system in the preclinical stages. However, it is very difficult to explain how therapeutic agents are distributed and how they interact with the biological environment, and their effect on cell death based on *in vivo* models. The mission of mathematical models is to investigate different methods and optimize them. This will reduce errors and the number of tests of in vitro and in vivo models, as well as reduce their costs; therefore, the clinical translation of successful will also be accelerated. The present study, as such, has developed a mathematical model that predicts the success of the current drug delivery system by considering the factors affecting the transport of therapeutic agents and their interactions. Due to the complexity of the proposed drug delivery system, the mathematical model includes detailed equations and parameters that are influenced by therapeutic conditions, temperature, and other functions. In the present study, it was found that if the size of the primary and secondary nanoparticles is optimally selected to control nano–bio interactions, which can suppress the proliferation of cancer cells for a long time. Therefore, the proposed mathematical model is a step forward in achieving targeted oncology and can be used for prediction in drug delivery processes.

## Supplementary Material

Supplemental Material

## Data Availability

The data supporting this work are accessible upon reasonable request from the corresponding author.
