# Supplementary material for: Stimuli-sensitive nano-drug delivery with programmable size changes to enhance accumulation of therapeutic agents in tumors
Source: Drug Deliv. 2023 Mar 9;30(1):2186312. doi: 10.1080/10717544.2023.2186312 (PMC10013474; doi:10.1080/10717544.2023.2186312)
Supplement: Supplemental Material [file IDRD_A_2186312_SM8290.zip › Supplementary Video and File/Supplementary file (1).docx]

**Supplementary file**

**Stimuli-sensitive nano-drug delivery with programmable size changes to enhance accumulation of therapeutic agents in tumors**

**Mohammad Souri et al.**

This document provides a detailed description of the mathematical model. Finally, the parameters that are affected by various factors such as temperature are investigated.

1. **Angiogenesis model**

The present angiogenesis model is inspired by the sprouting angiogenesis model initially proposed by Anderson and Chaplin [1, 2]. The mathematical model predicts capillary formation by tracking the motion of endothelial cells (ECs) located at the tip of the capillary sprout, ultimately forming a vascular network. The details of the rules and algorithm of the sprouting angiogenesis model have been determined in the previous publications [1, 2]. In summary, three main mechanisms for the motion of ECs are considered in this model: random motility, chemotaxis, and haptotaxis. Each of the motion terms can be described with the corresponding gradient of the motion stimuli and the appropriate coefficients. Random motility is modeled like diffusion of mass due to the concentration gradient. Chemotaxis is related to the concentration gradient of tumor angiogenesis factor (TAF). Haptotaxis is the motion of cells due to the adhesion gradient of bound fibronectin in the environment. The general system of equations describing the interactions between EC density ($n$), fibronectin ($f$) and TAF ($c_{TAF}$), are respectively written in Eqs. S1–S3 as [1, 2]:

| $\frac{\partial n}{\partial t}=\underset{random motility}{\underbrace{D_{n}\nabla^{2}n}}-\underset{chemotaxis and haptotaxis}{\underbrace{\nabla\cdot[\chi\left( 1+\delta c_{TAF} \right)n\nabla c_{TAF}+\phi n\nabla f]}}$ | (S1) |
| --- | --- |
| $\frac{\partial f}{\partial t}=\underset{production}{\underbrace{\beta n}}-\underset{uptake}{\underbrace{\gamma nf}}$ | (S2) |
| $\frac{\partial c_{TAF}}{\partial t}=\underset{uptake}{\underbrace{-\eta nc_{TAF}}}$ | (S3) |

$D_{n}$ is the random motility coefficient of EC, $\chi$ is the chemotaxis coefficient, $\delta$ is constant of chemotaxis coefficient, $\phi$ is haptotaxis coefficient, $\beta$ and $\gamma$ are production and uptake coefficient of fibronectin, respectively, and $\eta$ is uptake coefficient of TAF by ECs.

**2. Flow simulation in capillary network**

**2.1. Intravascular and interstitial fluid flow**

Blood flow in microvascular network is solved by finding pressure at interconnecting points in a network [3]. The flow rate in each vessel is calculated by applying mass conservation at each network junction. For intravascular blood flow, the continuity equation at an interconnecting point in the network, like c, is written as Eq. S4. In this equation, $Q_{c}^{k}$ is the net blood flow rate for each capillary calculated as the difference between the intravascular blood flow rate ($Q_{b.c}^{k}$), and the transvascular fluid flow rate ($Q_{t.c}^{k}$). The Reynolds number for blood flow in the capillary network is very low. Therefore, Poissville's law can be applied for $Q_{b.c}^{k}$ (Eq. S5) [3]. $Q_{t.c}^{k}$ is calculated by Starling's law as written in Eq. S6 [3].

| (S4) | $\sum_{k=1}^{N} Q_{c}^{k}\beta_{k}=0$, $Q_{c}^{k}=Q_{b,c}^{k}-Q_{t,c}^{k}$ |
| --- | --- |
| (S5) | $Q_{b,c}^{k}=\frac{\pi}{128}\frac{\Delta P_{b}d^{4}}{l \mu(d,H)}$ |
| (S6) | $Q_{t,c}^{k}=\pi dlL_{p}[\bar{P}_{b}-\bar{P}_{i}]$ |

In Eq. 4, the index $k$ refers to adjacent nodes, and $N$ is the number of peripheral vessel lattice nodes adjacent to the central vessel node. In the 2-D simulation for a fully connected network, $N$ is 4 and $\beta_{k}$ is a positive integer '0' or '1', which describes whether nodes k and c are connected ($\beta_{k}$= 1) or not ($\beta_{k}$= 0). In Eq. S5, $P_{b}$ is intravascular pressure (IVP), H is hematocrit, and $d$ and $l$ are the diameter and length of the new vessel, respectively. In Eq. 6, $L_{p}$ is the hydraulic conductivity of the microvascular wall, $\bar{P}_{b}$ is the average IVP in each element, $\bar{P}_{i}$ is the average interstitial fluid pressure (IFP) outside of the vascular element, $\pi_{b}$ is the osmotic pressure of the intravascular plasma, $\pi_{i}$ is the osmotic pressure of the interstitial fluid, and $\sigma$ is the average osmotic reflection coefficient for plasma proteins.

| 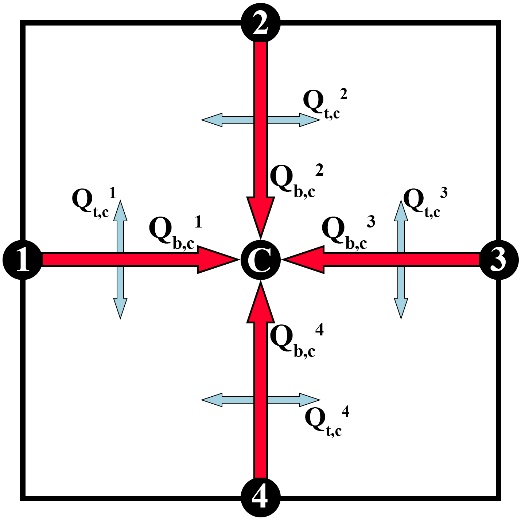 |
| --- |
| Schematic of intravascular and transvascular flow in an interconnecting point. |

IVP is calculated by applying Eq. S5. In contrast, IFP is calculated by solving the governing equation for fluid flow through a porous medium. The mass balance equation for a steady-state incompressible fluid with source and sink terms for biological tissues is written in Eq. S7. Blood vessels are fluid source terms, and lymphatic vessels are sink terms, which are respectively shown in Eqs. S8 and S9. In addition, Darcy’s observations show that the interstitial fluid velocity (IFV) in porous media is proportional to the pressure gradient (Eq. S10). By combining Eq. S7 and Eq. S10, the Eq. S11 can have derived to calculate IFP in both tumor and normal tissues.

| $\nabla\cdot v_{i}=F_{v}-F_{ly}$ | (S7) |
| --- | --- |
| $F_{v}=\frac{L_{P}S}{V}\left( \bar{P}_{b}-\bar{P}_{i} \right)$ | (S8) |
| $F_{ly}=\frac{L_{PL}S_{L}}{V}(\bar{P}_{i}-P_{L})$ | (S9) |
| $v_{i}=-K\nabla P_{i}$ | (S10) |
| $-{K\nabla}^{2}P_{i}=\left\{ \begin{aligned} \left\{ \begin{aligned} F_{v}-F_{ly} \\ F_{v} \end{aligned} \right. \\ \left\{ \begin{aligned} -\phi_{L} \\ 0 \end{aligned} \right. \end{aligned} \right. \begin{matrix} \begin{matrix} Normal tissue \\ Tumor tissue \end{matrix} & For existence blood source \\ \begin{matrix} Normal tissue \\ Tumor tissue \end{matrix} & Otherwise \end{matrix}$ | (S11) |

$v_{i}$ is the IFV, $F_{v}$ is the rate of fluid flow per unit volume from blood vessels into the interstitial space, $F_{ly}$ is the rate of fluid flow per unit volume from the interstitial space into lymph vessels, $S/V$ is the surface area of the microvascular per unit volume for mass transport in the interstitial space, $P_{L}$ the hydrostatic pressure of the lymphatic, and $K$ is the hydraulic conductivity of the interstitial space.

**2.2. Hemorheology**

Blood has significant non-Newtonian properties in low Reynolds numbers. The dynamic viscosity of blood ($\mu_{blood}$) in capillaries is shown in Eq. S12 [4] where $\mu_{plasma}$ and $\mu_{rel}$ are the dynamic viscosity of plasma and apparent viscosity of blood, respectively. The apparent blood viscosity is defined by Eq. S13. $\mu_{0.45}$ is the relative apparent blood viscosity for a fixed hematocrit of 0.45, which is defined in Eq. S14.

| $\mu_{blood}=\mu_{rel}\cdot\mu_{plasma}$ | (S12) |
| --- | --- |
| $\mu_{rel}=\left[ 1+\left( \mu_{0.45}-1 \right)\frac{\left( 1-H \right)^{C}-1}{\left( 1-0.45 \right)^{C}-1}\left( \frac{d}{d-1.1} \right)^{2} \right]\left( \frac{d}{d-1.1} \right)^{2}$  $C=\left( 0.8+\exp\left( -0.075d \right) \right)\left[ \frac{1}{1+{10}^{-11}d^{12}}-1 \right]+\frac{1}{1+{10}^{-11}d^{12}}$ | (S13) |
| $\mu_{0.45}=3.2+6\exp\left( -0.085d \right)-2.44\exp\left( -0.06d^{0.645} \right)$ | (S14) |

Hematocrit distribution at vessel bifurcations can change depending on the flow velocity in each branch. In other words, if the velocity ratio of the two branches exceeds the threshold value ($U_{cr}$), all hematocrit enter the faster branch at bifurcations [5]. The relation between the hematocrit of the parent vessel ($H_{i}$), and the branches, $H_{1}$ and $H_{2}$, are written as Eqs. S15-S17, respectively, based on the velocity ratio of the two branches, $U_{1}/U_{2}$. $\alpha$ is a phenomenological parameter that accounts for the strength of the non-symmetry of the hematocrit distribution at bifurcations [5].

| $H_{i}=H_{1}+H_{2}$ , $U_{1}>U_{2}$ | (S15) |
| --- | --- |
| $if \frac{U_{1}}{U_{2}}>U_{cr} \to\left\{ \begin{aligned} H_{1}=H_{i} \\ H_{2}=0 \end{aligned} \right.$ | (S16) |
| $if \frac{U_{1}}{U_{2}}<U_{cr} \to\frac{H_{1}}{H_{2}}=\alpha\frac{U_{1}}{U_{2}}$ | (S17) |

1. **Bioheat transfer**

In localized heating in tumor therapy, both blood vessels and tissue including the tumor and its holding tissue are heated. Energy balances for tissue and blood are illustrated as:

| 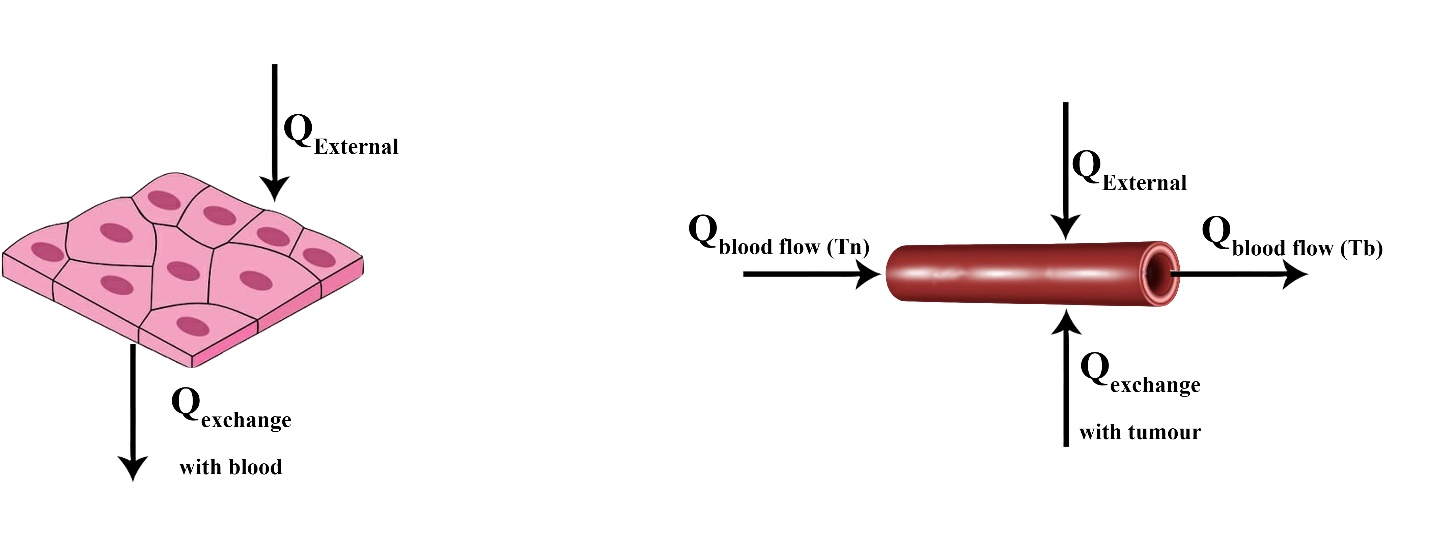 |
| --- |
| Schematic representation of heat transfer under external stimuli tissue and blood. |

The temperature (T) of tissue and blood can be calculated by solving the following energy balance equations [6-8]:

| $\rho_{t}c_{t}\frac{\partial T_{t}}{\partial t}={k_{t}\nabla}^{2}T_{t}-\underset{The heat sink term due to blood perfusion}{\underbrace{\rho_{bl}c_{bl}w_{bl}\left( T_{t}-T_{b} \right)}}+\underset{The heat generated by metabolism}{\underbrace{Q_{m}}}+\underset{The external power deposition term}{\underbrace{Q_{x}}}$ | (S18) |
| --- | --- |
| $\rho_{bl}c_{bl}\frac{\partial T_{b}}{\partial t}={k_{bl}\nabla}^{2}T_{bl}-\underset{The heat sink term due to blood perfusion}{\underbrace{\rho_{bl}c_{bl}w_{bl}\left( T_{b}-T_{n} \right)}}+\underset{The heat source term due to tissue}{\underbrace{\rho_{bl}c_{bl}w_{bl}\left( T_{t}-T_{b} \right)}}+\underset{The heat generated by metabolism}{\underbrace{Q_{m}}}+\underset{The external power deposition term}{\underbrace{Q_{x}}}$ | (S19) |

Where $\rho_{t}$, $c_{t}$ and $k_{t}$ are the density, heat capacity and thermal conductivity of the tissue, respectively. $\rho_{bl}$, $c_{bl}$ and $k_{bl}$ are the density, heat capacity and thermal conductivity of blood, respectively. $w_{bl}$ is the perfusion rate of blood flow.$Q_{x}$ is the heat produced by an external stimulus. In this study, external stimuli is focused ultrasound.

1. **Focused ultrasound-mediated hyperthermia**

Focused ultrasound therapy is a noninvasive ablation method in which ultrasound energy from an extracorporeal source is focused within the body to locally ablate tissue at the focus without damaging surrounding tissues [9, 10]. Focused ultrasound is most widely used to thermally ablate a variety of both benign and malignant tumors including uterine fibroids, prostate cancer, breast cancer, liver tumors, and other solid tumors that are accessible to ultrasound energy [11-13]. In present study, the propagation of ultrasound in a medium is investigated by solving linear propagation of the pressure wave is given by the Helmholtz equation [14]:

| $\frac{1}{\rho c_{0}^{2}}\frac{\partial^{2}P}{\partial t^{2}}+\nabla\cdot\left[ -\frac{1}{\rho}\left( \nabla P-q_{d} \right) \right]=Q_{p}$ | (S20) |
| --- | --- |

Where $\rho$ and $c_{0}$ are the density and the speed of sound, respectively. $Q_{p}$ and $q_{d}$ are possible acoustic monopole and dipole source terms, respectively, which are considered zero. $Q_{p}$ can be used to represent a domain heat source causing pressure variations or a user defined nonlinear contribution to the equations in the time domain (Westervelt equation [8]). $q_{d}$ source represents a domain volumetric force.

The pressure, P, can be expanded into harmonic components using the Fourier series:

| $P=psin\left( \omega t \right)==\gg P=pe^{-i\omega t}$ | (S21) |
| --- | --- |

Solving Eq. S21 with this complex variable yields the Helmholtz equation:

| $\frac{\kappa^{2}}{\rho}p+\nabla\cdot\left[ \frac{1}{\rho}\left( \nabla p \right) \right]=0$ | (S22) |
| --- | --- |

where $\kappa$ is the wave number that it's defined by;

| $\kappa=\frac{\omega}{c}+i\alpha_{ABS}$ | (S23) |
| --- | --- |

here $\alpha_{ABS}$ is the absorption coefficient. $\omega(=2\pi f)$ is the angular frequency and $f$ is the frequency.

The power ($P_{W}$) of the acoustic wave has a relation with the transducer's operating parameters through Eq. (S24) [10]:

| $P_{W}=2S\pi^{2}\rho_{w}fc_{w}{\Delta x}_{max}^{2}$ | (S24) |
| --- | --- |

where $S$ is the transducer area, $\rho_{w}$ and $c_{w}$ are the density and the speed of sound of the medium that is in contact with the transducer aperture, here is the water between the transducer and the tissue. $\Delta x$ is the normal displacement of the transducer aperture and the transducer contact area oscillates harmonically with this displacement value and transmits the desired sound pressure. Therefore, the transducer power could be regulated by changing the amount of normal displacement.

In order to couple the pressure field to the temperature field, we need to estimate the thermal energy deposition associated with the absorption of ultrasonic waves. The following equation [15] describes the ultrasonic power deposition per unit volume:

| $Q_{x}=2\alpha_{ABS}I=2\alpha(\frac{P^{2}}{2\rho})$ | (S25) |
| --- | --- |

1. **Therapeutic agents transport**

Cytotoxic drugs are encapsulated in secondary nanoparticles and secondary nanoparticles are encapsulated in primary nanoparticles to reduce the risk of effects caused by high concentrations of smaller nanoparticles and drugs in healthy tissues, then administered intravenously into the bloodstream. When systematically administered, NPs are delivered to tumor regions via the circulatory system. In the following, the mathematical equations governing the transport of therapeutic agents, release operations, diffusion of therapeutic agents in the tissue, and their exchange between biological environments are presented.

- 1. **Primary nanoparticles**

Pharmacokinetics of primary nanoparticles is described by one compartment, i.e., systemic plasma. Following an IV injection (Initial concentration of primary nanocarrier: $40 \mu g/ml$), the concentration of primary nanoparticles in the systemic plasma compartment ($\partial C_{N1C}$) is determined by [16]:

| $V_{Sp}\frac{\partial C_{N1C}}{\partial t}=-{CL}_{N1}C_{N1C}V_{Sp}-{kr}_{37}C_{N1C}V_{Sp}-F_{PT}C_{N1C}V_{Tp}+F_{PT}C_{N1M}V_{Tp}$ | (S26) |
| --- | --- |

where${CL}_{N1}$, ${kr}_{37}$ are the clearance rate of primary nanoparticles and release rate (at 37$℃$) in the systemic plasma, respectively. $V_{Sp}$, $V_{Tp}$ and $F_{PT}$ are the volume of systemic plasma, volume of microvessel plasma and plasma flow per microvessel plasma volume, which are functions of hematocrit and are defined as the following Eqs.

| $V_{Sp}=V_{BB}(1-H_{ct})$ | (S27) |
| --- | --- |
| $V_{Tp}= V_{T}\times v_{Tp}$ | (S28) |
| $v_{Tp}=$($V_{TV}(1-H_{ctt})$) | (S29) |
| $F_{PT}=\omega(1-H_{ctt})/v_{Tp}$ | (S30) |

Here $V_{BB}$, $H_{ct}$, $H_{ctt}$, $V_{T}$, $V_{TV}$ and $\omega$ are total blood volume in body, Hematocrit, Hematocrit for tissue microvasculature, volume of tissue, volume fraction of tissue vascular space, and blood perfusion rate, respectively.$v_{Tp}$ is volume fraction of microvessel plasma space.

The concentration of primary nanoparticles in microvessels is defined as:

| $V_{Tp}\frac{\partial C_{N1M}}{\partial t}=-E_{N1M}C_{N1M}V_{Tp}-{kr}_{T}C_{N1M}V_{Tp}+F_{PT}C_{N1C}V_{Tp}-F_{PT}C_{N1M}V_{Tp}$ | (S31) |
| --- | --- |

where$E_{N1M}$, ${kr}_{T}$ are the elimination rate of primary nanoparticles and release rate under mild hyperthermia, respectively.

- 1. **Secondary nanoparticle**

Secondary nanoparticles are released due to the instability of the primary nanoparticles at a very slow-release rate at body temperature. The concentration of secondary nanoparticles in the systemic circulation is defined as follows:

| $V_{Sp}\frac{\partial C_{N2C}}{\partial t}=-{CL}_{N2}C_{N2C}V_{Sp}-F_{PT}C_{N2C}V_{Tp}+F_{PT}C_{N2M}V_{Tp}$ | (S32) |
| --- | --- |

here${CL}_{N2}$ is the clearance rate of secondary nanoparticles.

The release rate of secondary nanoparticles in circulation is zero due to their high stability. The concentration of secondary nanoparticles in the microvascular network is defined according to Eq. S37. The exchange of secondary nanoparticles between the microvascular network and interstitium is defined based on the pore model (Eq. S38).

| $V_{Tp}\frac{\partial C_{N2M}}{\partial t}=-E_{N2M}C_{N2M}V_{Tp}-{Ex}_{pore}V_{Tp}+F_{PT}C_{N2C}V_{Tp}-F_{PT}C_{N2M}V_{Tp}$ | (S33) |
| --- | --- |
| ${Ex}_{pore}=\left( F_{v}\left( 1-\sigma_{N2} \right)C_{N2M}+P_{N2}\frac{S}{V}\left( C_{N2M}-C_{N2I} \right)\frac{{Pe}_{N2}}{e^{{Pe}_{N2}}-1} \right)$ | (S34) |

Here $E_{N2M}$ is the elimination rate of secondary nanoparticles. $\sigma_{N2}$ and $P_{N2}$ are the osmotic reflection coefficient for the secondary nanoparticles, and the permeability of microvascular wall. Also, ${Pe}_{N2}$ is the trans-capillary Peclet number for secondary nanoparticles that it’s defined as:

| ${Pe}_{N2}=\frac{F_{v}\left( 1-\sigma_{N2} \right)}{P_{L}\frac{S}{V}}$ | (S35) |
| --- | --- |

The distribution of secondary nanoparticles in the interstitium is defined based on diffusion and convection mechanisms. Also, the secondary nanoparticles in the interstitium release their cargo at a certain rate in response to the acidity.

| $\frac{\partial C_{N2I}}{\partial t}+\nabla\cdot\left( C_{N2I}v_{i} \right)=D_{N2}\nabla^{2}C_{N2I}-{kr}_{pH}C_{N2I}+{Ex}_{pore}$ | (S36) |
| --- | --- |

here $D_{N2}$ and ${kr}_{pH}$ are the diffusion coefficient and release rate in response to pH, respectively.

- 1. **Free drug**

Free drugs are released from secondary nanoparticles accumulated in the interstitium and distributed according to diffusion and convection mechanisms. Various factors such as exchange with intracellular, association/disassociation with protein, escape from interstitium affect the concentration of free drug in the interstitium. The concentration of free drug in the interstitium is defined as follows:

| $\frac{\partial C_{FI}}{\partial t}+\underset{\mathrm{Convection}}{\underbrace{\nabla\cdot\left( C_{FI}v_{i} \right)}}=\underset{\mathrm{Diffusion}}{\underbrace{D_{F}\nabla^{2}C_{FI}}}+{kr}_{pH}C_{N2I}-\left( P_{F}\frac{S}{V}\left( C_{FI} \right) \right)+\left( k_{d}C_{BI}-k_{a}C_{FI} \right)+\left( D_{c}\varepsilon-D_{C}\zeta\right)$ | (S37) |
| --- | --- |

where $D_{F}$ is the diffusion coefficient of free drugs. $k_{a}$ and $k_{d}$ are the protein binding and dissociation rates, respectively.$D_{c}$ is the tumor cell density and ζ and ɛ are cellular uptake and efflux functions.

The concentration of free drugs in the microvascular network (Eq. S42) and systemic circulation (Eq. S43) is as follows;

| $V_{Tp}\frac{\partial C_{FM}}{\partial t}=+\left( P_{L}\frac{S}{V}\left( C_{FI} \right) \right)V_{Tp}+(k_{d}C_{BM}-k_{a}C_{FM})V_{Tp}-E_{FM}C_{FM}V_{Tp}-F_{PT}C_{FM}V_{Tp}$ | (S38) |
| --- | --- |
| $V_{Sp}\frac{\partial C_{FC}}{\partial t}=({k_{d}C_{BC}-k}_{a}C_{FC})V_{Sp}+{(k_{ft}C_{FC}V_{BT}-k}_{fP}C_{FC}V_{Sp})-{CL}_{F}C_{FC}V_{Sp}+F_{PT}C_{FM}V_{Tp}$ | (S39) |

Here $E_{FM}$ and ${CL}_{F}$ are the elimination rate and is the clearance rate free drugs, respectively. $k_{fp}$ and $k_{ft}$ are transport constant drug from systemic plasma to body tissue and transport constant drug from body tissues to systemic plasma, respectively. $V_{BT}$ is the volume of body tissue.

- 1. **Bound drug**

The distribution of the bound drug in the different compartments is the same as that of the free drug, except that it does not include release and cell exchange terms in the interstitium.

| $\frac{\partial C_{BI}}{\partial t}+\underset{\mathrm{Convection}}{\underbrace{\nabla\cdot\left( C_{BI}v_{i} \right)}}=\underset{\mathrm{Diffusion}}{\underbrace{D_{B}\nabla^{2}C_{BI}}}-\left( P_{B}\frac{S}{V}\left( C_{BI} \right) \right)-\left( k_{d}C_{BI}-k_{a}C_{FI} \right)$ | (S40) |
| --- | --- |
| $V_{Tp}\frac{\partial C_{BM}}{\partial t}=+\left( P_{B}\frac{S}{V}\left( C_{BI} \right) \right)V_{Tp}-(k_{d}C_{BM}-k_{a}C_{FM})V_{Tp}-E_{BM}C_{BM}V_{Tp}-F_{PT}C_{BM}V_{Tp}$ | (S41) |
| $V_{Sp}\frac{\partial C_{BC}}{\partial t}=-({k_{d}C_{BC}-k}_{a}C_{FC})V_{Sp}+{(k_{bt}C_{BC}V_{T}-k}_{bP}C_{BC}V_{Sp})-{CL}_{B}C_{BC}V_{Sp}+F_{PT}C_{BM}V_{Tp}$ | (S42) |

- 1. **Intracellular concentration**

Since only unbound drug can pass through the cell membrane [17, 18], the rate of cellular uptake is a function of free drug concentration in the interstitial fluid [7].

| $\frac{\partial C_{i}}{\partial t}=V_{max}\frac{C_{fe}}{C_{fe}+k_{e}\varphi}-V_{max}\frac{C_{i}}{C_{i}+k_{i}}$ | (S43) |
| --- | --- |

where $V_{max}$ is the rate of transmembrane transport, ζ and ɛ are cellular uptake and efflux functions due to multidrug resistance pumps, $k_{e}$ and $k_{i}$ are constants obtained from experimental data, and φ is the volume fraction of extracellular space.

1. **Pharmacodynamics model**

The change of tumor cell density with time is described by a pharmacodynamics model as given below [19]. This model is defined based on intracellular concentration over time and define as:

| $\frac{dD_{c}}{dt}=-\frac{f_{max}C_{i}}{{EC}_{50}+C_{i}}D_{c}+k_{c}D_{c}-k_{g}D_{c}^{2}$ | (S44) |
| --- | --- |

The first term on the right-hand side represents the anticancer effect, where $f_{max}$ is the cell-kill rate constant and ${EC}_{50}$ is the drug concentration producing 50% of fmax. $k_{c}$ and $k_{g}$ are cell proliferation rate constant and physiological degradation rate, respectively. In this study, cell proliferation and physiologic degradation are assumed to have reached equilibrium at the start of each treatment.

1. **Oxygen Concentration**

The rate of change of oxygen in tissues depends on its transport through convection and diffusion, minus the amount of oxygen consumed by cells, plus the amount that enters the tissue from the blood vessels:

| $\frac{dC_{ox}}{dt}+\nabla\cdot\left( C_{ox}v^{f} \right)=D_{ox}\nabla^{2}C_{ox}-\frac{A_{ox}C_{ox}}{C_{ox}+k_{ox}}\frac{D_{c}}{D_{C TOTAL}}+P_{er}\frac{S}{V}\left( C_{iox}-C_{ox} \right)-k_{g}D_{c}^{2}$ | (S45) |
| --- | --- |

where $C_{ox}$ is the oxygen concentration, $D_{ox}$ is the diffusion coefficient of oxygen in the interstitial space, $A_{ox}$ and $k_{ox}$ are oxygen uptake parameters, $P_{er}$ is the vascular permeability of oxygen that describes diffusion across the tumor vessel wall and Ciox is the oxygen concentration in the vessels.

1. **Model parameters**

Values adopted for modeling are summarized in tables at the end of this chapter for parameters related to the physiological parameters, parameters for doxorubicin, parameters for nanoparticles. Justifications for the choices of some of the parameters are given below. Since temperature elevations in response to heating may influence some of the properties used in the drug transport model, temperature dependence of these properties is also considered.

- 1. **Tissue related transport parameters**
- **Perfusion rate**

Spatial distribution of blood perfusion is considered like vascular density. On the other hand, blood perfusion rate ($\omega$) also depends on temperature [20] by the following relation:

| $\omega=\omega_{0}e^{-\int_{0}^{t} Ae^{\frac{-\Delta E}{RT(t)}}dt}$ | (S46) |
| --- | --- |

Where $\omega_{0}$ represents the time dependent perfusion at $37˚C$. $R$ is the universal gas constant, $A$ is a frequency factor for the kinetic expression, and $\Delta E$ is the activation energy of the thermal damage process, and $T$ is the instantaneous absolute temperature of the cells during thermal stress, which is a function of time, $t$. The parameters $A$ and $\Delta E$ are dependent on the type of tissue and have been characterized for normal breast tissues by Henriques and Moritz [21] and breast tumor tissues by Bhowmik et al [22].

- **Thermal conductivity**

Thermal conductivity like other parameters varies with temperature, which is defined as follows [23]:

| $k\left( t \right)=k_{0}+0.001161(T-T_{0})$ | (S47) |
| --- | --- |

Where $k_{0}$ is the thermal conductivity at $T_{0}$, that $T_{0}$ is $37˚C$.

- **Absorption coefficient**

The absorption coefficient increases by the frequency enhancement and could be calculated by Eq. S48 [10]:

| $\alpha_{ABS}=\alpha_{0}\left( \frac{f}{f_{0}} \right)^{\beta}$ | (S48) |
| --- | --- |

where $\beta$ is the absorption power, and $\alpha_{0}$ is the absorption coefficient at $f_{0} = 1 MHz$. The absorption coefficient changes linearly with the frequency for biological soft tissues [24], therefore, $\beta$ = 1.

- 1. **Drug related transport parameters**
- **Vascular permeability**

Vascular permeability coefficient measures the capacity of a microvessel wall to allow for the flow of substances in and out of the vasculature. The structure of microvessel wall and the molecular size of the transported substance are key determinants of permeability [25].

- Free & bound drug

Estimates of this parameter reported in the literature usually correspond to ‘effective permeability’, which is on the order of ${10}^{-7} m/s$ for albumin in both tumor and normal tissues. Compared with normal tissues, Gerlowski and Jain [26] found the vessel wall permeability to be 8 times higher in tumor tissues. Drug permeability variation based on temperature is logarithmic [7]. Hence, Zhan [7] estimated the fold increase in permeability for free and bound drug as follows:

| $\frac{P_{0}}{P}={10}^{0.7(T_{0}-T_{2})}$ | (S49) |
| --- | --- |

Where $P_{0}$ and $P$ are the permeability of Free and bound drug at $T_{0}$ and $T$, respectively.

- Nanoparticles

For a baseline temperature of 34 ˚C, extracellular concentrations of nanoparticle were found to have increased by 76-fold and 38-fold upon heating to 45˚C and 42˚C, respectively [27]. Based on these experimental data [27], a relationship between fold increase in permeability and temperature defined [7].

| $\frac{P}{P_{0}}=-10.54+5.76e^{\left( \frac{T-T_{0}}{5.44} \right)}+5.78e^{\left( \frac{T-T_{0}}{5.46} \right)}$ | (S50) |
| --- | --- |

where $P_{0}$ and $P$ are the permeability of nanoparticle at $T_{0}$ and $T$, respectively.

- **Diffusion coefficient**

Diffusion coefficient is the constant of proportionality between the particle flux owing to particle diffusion and the gradient in the concentration of the species, which is also known as the driving force for diffusion.

- Free & bound drug

Diffusion coefficient is related to the molecular weight (MW) of the Dox [15], Temperature does not have a significant effect on the diffusion coefficient of Free and bound drug.

- Nanoparticle

According to the Stokes-Einstein equation, diffusivity based on temperature and viscosity. So, the fold increase in diffusivity of nanoparticle as a function of temperature and viscosity are [7]:

| $\frac{D_{0}}{D}=\frac{T_{0}\mu}{T\mu_{0}}$ | (S51) |
| --- | --- |

where $D_{0}$ and $D$ are the diffusivity coefficient nanoparticle at $T$ and $T_{0}$, respectively. Owing to the lack of relevant data, viscosity values are assumed to be those of water [28], whose dependence on temperature is given by Eq. S52 [7]. So, the fold increase in diffusivity of viscosity is given by Eq. S53 [7].

| $\mu_{w}=exp(5.1-0.03T+1.04\times{10}^{-4}T^{2})$ | (S52) |
| --- | --- |
| $\frac{\mu}{\mu_{0}}=exp(-0.03\left( T-T_{0} \right)+1.04\times{10}^{-4}(T^{2}-T_{0}^{2}))$ | (S53) |

- **Transmembrane rate**

The transmembrane parameter was determined by El-Kareh and Secomb [17] by curve fitting to data obtained from in vitro experiments [29]. It has been suggested that increased cellular uptake of Dox with heating will lead to the improved outcomes when the drug is administrated simultaneously with hyperthermia [17]. Based on data in [30], there is a 2.2-fold increase at 42˚C. Here, the fold increase at temperature $T$ is obtained by linear interpolation [7].

| $k_{tv}=0.24T-7.88$ | (S54) |
| --- | --- |

- 1. **Drug release**

Explosive release rates of secondary nanoparticles from primary nanoparticles in response to temperature based on the intravascular release paradigm have not been reported. While sufficient information is available to estimate the drug release rate. Therefore, in the present study, the release rate of secondary nanoparticles is assumed like drug release rate, which is defined as follows in the previous study [31];

| Release rates at various temperatures | | | | | |
| --- | --- | --- | --- | --- | --- |
| $T (℃)$ | 37 | 38 | 39 | 40 | 41.3 |
| $\mathrm{kr}_{T}$ | 0.0003 | 0.0047 | 0.142 | 0.221 | 0.3 |

Also, at neutral pH, the drug release rate is considered zero; so, there is no free drug in the circulatory system and microvascular network. According to the previous study [32], the drug release rate from secondary nanoparticles at different pH level is as follows;

| Release rates at various pH levels | | | | | | |
| --- | --- | --- | --- | --- | --- | --- |
| $pH$ | 6.2 | 6.4 | 6.6 | 6.8 | 7 | 7.2 |
| $\mathrm{kr}_{pH}$ | $5.71\times{10}^{-5}$ | $5.59\times{10}^{-5}$ | $5.48\times{10}^{-5}$ | $5.37\times{10}^{-5}$ | $5.26\times{10}^{-5}$ | $5.15\times{10}^{-5}$ |
|  |  |  |  |  |  |  |

| Table. S1. The parameters used for Angiogenesis model and Flow simulation in capillary network. | | | |
| --- | --- | --- | --- |
| **Parameters** | **Description** | **Value** | **References** |
| **Angiogenesis model** | | | |
| $D_{n}$ | Random motility coefficient of EC | 3.5×10^-4^ | [1] |
| $\chi$ | Chemotaxis coefficient | 0.16 | [1] |
| $\delta$ | Constant of chemotaxis coefficient | 0.6 | [33] |
| $\phi$ | Haptotaxis coefficient | 0.34 | [1] |
| $\beta$ | Production coefficient of fibronectin | 0.01 | [1] |
| $\gamma$ | Uptake coefficient of fibronectin | 0.1 | [1] |
| $\eta$ | Uptake coefficient of TAF | 0.1 | [1] |
| **Intravascular and interstitial fluid flow** | | | |
| $K$ | Interstitial hydraulic conductivity | 6.41×10^-15^ $m^{2}/(Pa\cdot s)$ for normal tissue | [34, 35] |
|  |  | 31.1×10^-15^ $m^{2}/(Pa\cdot s)$ for tumor tissue |  |
| $L_{p}$ | Hydraulic conductivity of the microvascular wall | 0.27×10^-11^ $m/(Pa\cdot s)$ for normal tissue | [36, 37] |
|  |  | 2.1×10^-11^ $m/(Pa\cdot s)$ for tumor tissue |  |
| $\sigma$ | Average osmotic reflection coefficient | 0.91 for normal tissue | [3, 38] |
|  |  | 0.82 for tumor tissue |  |
|  |  | 2000 $mmHg$ for tumor tissue |  |
| $S/V$ | Characteristic value of surface area per unit volume of capillaries for mass transport in the interstitial space | 7×10^3^ $m^{-1}$ for normal tissue | [3, 38] |
|  |  | 2×10^4^ $m^{-1}$ for tumor tissue |  |
| $\frac{L_{PL}S_{L}}{V}$ | Lymphatic filtration coefficient | 1.33×10^-5^ $\frac{1}{(pa\cdot s)}$ for normal tissue | [39] |
| $P_{L}$ | Hydrostatic pressure of lymphatic vessels | 0 $Pa$ | [39] |
| **Hemorheology** | | | |
| $U_{cr}$ | Threshold blood velocity ratio of two branches in bifurcations | 2.5 | [5] |
| $\alpha$ | Phenomenological parameter that accounts for the strength of the non-symmetry of the hematocrit distribution at bifurcations | 0.5 | [5] |
| $\mu_{plasma}$ | Dynamic viscosity of plasma | 1.2×10^-3^ $Pa\cdot s$ | [40] |
| **Remodeling capillary network** | | | |
| $\tau_{ref}$ | A positive constant as reference of wall shear stress | 0.103 $Pa$ | [4] |
| $k_{p}$ | Adaptive response sensitivity of the vessel diameter to changes in intravascular pressure | 0.1 $1/s$ | [33] |
| $k_{m}$ | Proportional coefficient of metabolic stimuli | 0.07 $1/s$ | [33] |
| $k_{s}$ | Shrinking tendency of the vessel in the absence of positive growth stimuli | 0.35 $1/s$ | [33] |

| Table S2. Physiological parameters. | | | | | | | |
| --- | --- | --- | --- | --- | --- | --- | --- |
| Parameter | Definition | Unit | Tumor Tissue | Normal Tissue | Blood | Whole  Body | Ref. |
| $\rho$ | Tissue density | $kg/m^{3}$ | 1000 | 1000 | 1060 | - | [35, 41] |
| $D_{c}$ | Cell density | ${10}^{5}cell/m^{3}$ | ${10}^{10}$ | ${10}^{10}$ | - | - | [7, 18] |
| $c_{0}$ | Ultrasound speed | $m/s$ | 1550 | 1550 | 1540 | - | [41] |
| $c$ | Specific heat | $J/(kg\cdot K)$ | 3800 | 3600 | 3770 | - | [41] |
| $k$ | Thermal conductivity | $W/(m\cdot K)$ | 0.552 | 0.512 | 0.53 | - | [41] |
| $w_{b0}$ | Blood perfusion rate at 37 $℃$ | $s^{-1}$ | 0.002 | 0.018 | - | - | [42] |
| $R_{g}$ | Universal gas constant | $J/(mol\cdot K)$ | 8.314 | 8.314 | - | - | [20] |
| $\Delta E$ | Activation energy | $J/mol$ | $6.67\times{10}^{5}$ | $6.67\times{10}^{5}$ | - | - | [20] |
| $A_{f}$ | Frequency factor | $s^{-1}$ | $1.98\times{10}^{106}$ | $1.98\times{10}^{106}$ | - | - | [20] |
| $V_{BB}$ | Total blood volume in body | l | - | - | - | 5.53 | [43] |
| $H_{ct}$ | Hematocrit | 1 | 0.19 | 0.45^*^ | - | 0.45 | [44, 45] |
| $V_{TV}$ | Volume fraction of tissue vascular space | 1 | 0.092 | 0.0322^*^ | - | - | [46] |
| $V_{BT}$ | Volume of body tissue | l | - | - | - | 64.47 | [43] |
| $Q_{m}$ | The heat generated by metabolism | $\frac{W}{m^{-3}}$ | 0 | 0 | - | - | [8, 47] |
| * The related values are assumed in this study. | | | | | | | |

| Table S3. Parameters for doxorubicin | | | | | |
| --- | --- | --- | --- | --- | --- |
| Parameter | Definition | Unit | Free Doxorubicin | Bound Doxorubicin | Ref. |
| $P_{tumor}$ | Permeability of vasculature wall in tumor tissue | $m/s$ | $3.6\times{10}^{-6}$ | $7.8\times{10}^{-9}$ | [48, 49] |
| $P_{\mathrm{normal}}$ | Permeability of vasculature wall in normal tissue | $m/s$ | $3.75\times{10}^{-7}$ | $2.5\times{10}^{-9}$ | [48, 49] |
| $D_{tumor}$ | Diffusion coefficient in interstitial fluid of tumor | $m^{2}/s$ | $3.4\times{10}^{-10}$ | $8.89\times{10}^{-12}$ | [48, 50-52] |
| $D_{\mathrm{normal}}$ | Diffusion coefficient in interstitial fluid of normal | $m^{2}/s$ | $1.58\times{10}^{-10}$ | $4.17\times{10}^{-12}$ | [48, 50-52] |
| $\sigma_{d}$ | Osmotic reflection coefficient | 1 | 0.15 | 0.82 | [53] |
| $k_{a}$ | Doxorubicin-protein binding rate | $s^{-1}$ | 0.833 | - | [18] |
| $k_{d}$ | Doxorubicin-protein dissociation rate | $s^{-1}$ | - | 0.278 | [18] |
| $\varphi$ | Tumor fraction extracellular space | 1 | 0.4 | - | [18] |
| $V_{max}$ | Rate of trans-membrane transport | $kg/{10}^{5}cell\cdot s$ | $4.67\times{10}^{-15}$ | - | [18, 29] |
| $k_{e}$ | Michaelis constant for transmembrane transport | $kg/m^{3}$ | $2.19\times{10}^{-4}$ | - | [18, 29] |
| $k_{i}$ | Michaelis constant for transmembrane transport | $kg/{10}^{5}cells$ | $1.37\times{10}^{-12}$ | - | [18, 29] |
| $f_{max}$ | Cell-kill rate constant | $s^{-1}$ | $1.67\times{10}^{-5}$ | - | [19] |
| ${EC}_{50}$ | Drug concentration producing 50% of $f_{max}$ | $kg/{10}^{5}cells$ | $5\times{10}^{-13}$ | - | [19] |
| $k_{c}$ | Cell proliferation rate | $s^{-1}$ | $3\times{10}^{-6}$ | - | [54] |
| $k_{g}$ | Cell physiologic degradation rate | $s^{-1}$ | $3\times{10}^{-16}$ | - | [54] |
| A | Parameter for pharmacokinetic model | $m^{-1}$ | 130 | - | [18, 55] |
| ${CL}_{Tumor}$ | Plasma clearance in tissue | $s^{-1}$ | $2.43\times{10}^{-3}$ | 0 | [56, 57] |
| ${CL}_{Sys}$ | Plasma clearance in Systemic | $s^{-1}$ | $1.1 \times{10}^{-3}$ | 0 | [58] |
| $k_{P}$ | Transfer constant free drug from systemic plasma to tissue | $s^{-1}$ | $1.6 \times{10}^{-3}$ | $9.6 \times{10}^{-5}$^*^ | [58] |
| $k_{t}$ | Transfer constant free drug from tissue to systemic plasma | $s^{-1}$ | $4.8 \times{10}^{-5}$ | $2.8 \times{10}^{-7}$^*^ | [58] |
| $t_{\frac{1}{2}}^{\alpha}$ | Half-life of doxorubicin in plasma | min | 4.75 | - | [55] |
| * The related values are assumed in this study. | | | | | |

| able S4. Parameters for liposome | | | | | | | |
| --- | --- | --- | --- | --- | --- | --- | --- |
| Parameter | Definition | Unit | Size (nm) | Tumor Tissue | Systemic | Ref. | |
| $P$ | permeability of vasculature wall | $m/s$ | 1 | $1.27\times{10}^{-8}$ | - | [59-63] | |
|  |  |  | 5 | $2.34\times{10}^{-9}$ | - |  |  |
|  |  |  | 12 | $9.5\times{10}^{-10}$ | - |  |  |
|  |  |  | 20 | $4.6\times{10}^{-10}$ | - |  |  |
|  |  |  | 150 | $0$ | - |  |  |
|  |  |  | 750 | 0 | - |  |  |
|  |  |  | 1040 | 0 |  |  |  |
| $D$ | Diffusion coefficient | $m^{2}/s$ | 1 | $3\times{10}^{-10}$ | - | [59-63] | |
|  |  |  | 5 | $8\times{10}^{-11}$ | - |  |  |
|  |  |  | 12 | $2.8\times{10}^{-11}$ | - |  |  |
|  |  |  | 20 | $7\times{10}^{-12}$ | - |  |  |
|  |  |  | 150 | 0 | - |  |  |
|  |  |  | 750 | 0 | - |  |  |
|  |  |  | 1040 | 0 |  |  |  |
| $\sigma$ | Reflection coefficient | 1 | 1 | 0.0001 | - | [47, 59-63] | |
|  |  |  | 5 | 0.00285 | - |  |  |
|  |  |  | 12 | 0.0098 | - |  |  |
|  |  |  | 20 | 0.043 | - |  |  |
|  |  |  | 150 | 1 | - |  |  |
|  |  |  | 750 | 1 | - |  |  |
|  |  |  | 1040 | 1 |  |  |  |
| $E$ | Elimination | $s^{-1}$ | 1 | $3.1\times{10}^{-2}$ | - | [59-64] | |
|  |  |  | 5 | $3.5\times{10}^{-3}$ | - |  |  |
|  |  |  | 12 | $4.3\times{10}^{-4}$ | - |  |  |
|  |  |  | 20 | $2.1\times{10}^{-4}$ | - |  |  |
|  |  |  | 150 | $2.228\times{10}^{-4}$ | - |  |  |
|  |  |  | 750 | $8.3\times{10}^{-4}$ | - |  |  |
|  |  |  | 1040 | $3.5\times{10}^{-2}$ |  |  |  |
| $CL$ | Clearance | $s^{-1}$ | 1 | - | $3.1\times{10}^{-2}$ | [43, 59-63] | |
|  |  |  | 5 | - | $3.5\times{10}^{-3}$ |  |  |
|  |  |  | 12 | - | $4.3\times{10}^{-4}$ |  |  |
|  |  |  | 20 | - | $2.1\times{10}^{-4}$ |  |  |
|  |  |  | 150 | - | $2.228\times{10}^{-4}$ |  |  |
|  |  |  | 750 | - | $8.3\times{10}^{-4}$ |  |  |
|  |  |  | 1040 |  | $3.5\times{10}^{-2}$ |  |  |
| 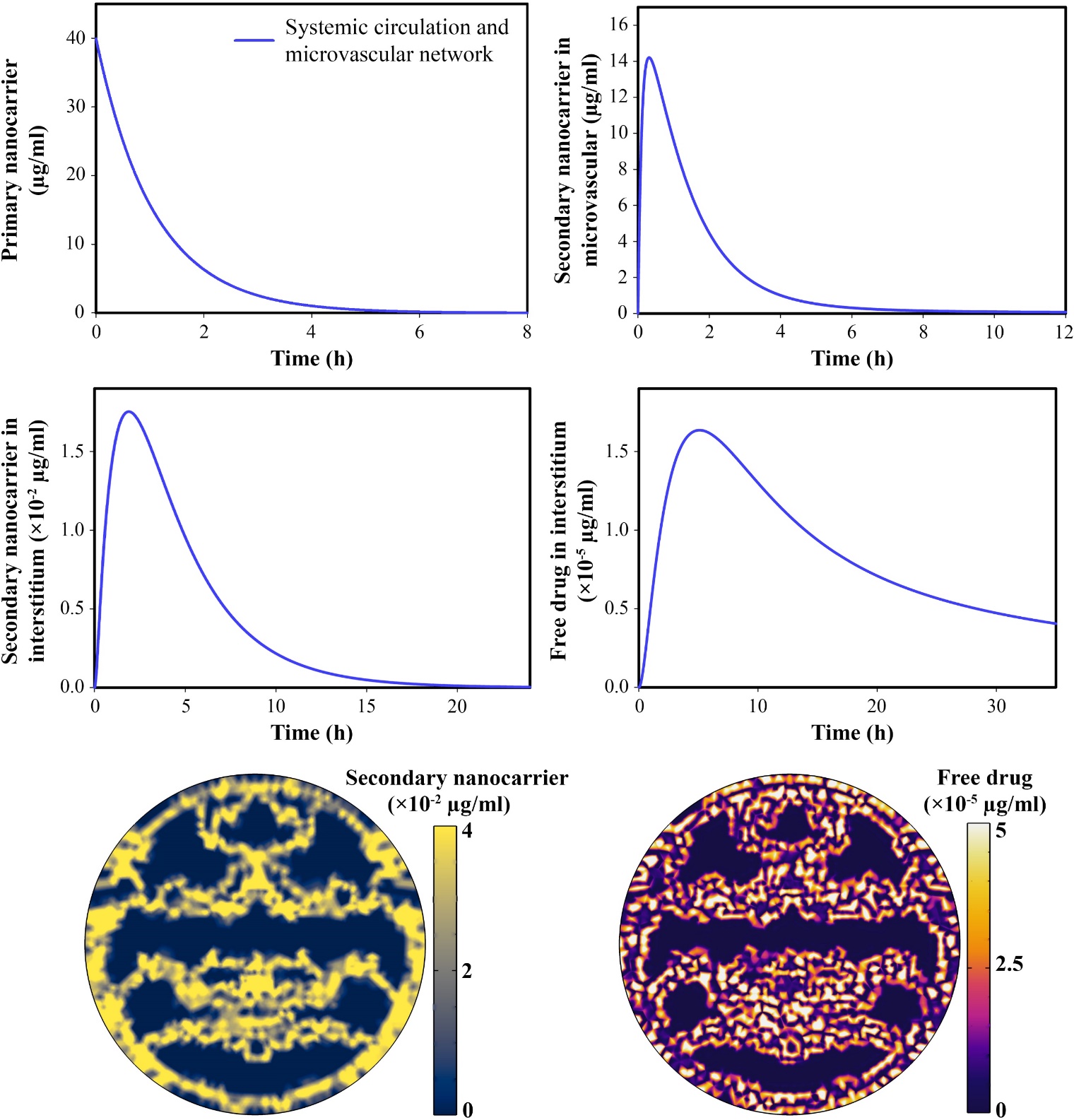 | | | | | | |  |
| Figure S1: In the absence of hyperthermia, secondary nanoparticles are released at a very weak rate at body temperature (due to the instability of the primary nanoparticles at body temperature). This does not provide a high concentration of therapeutic agents and high penetration depth. | | | | | | |  |

| 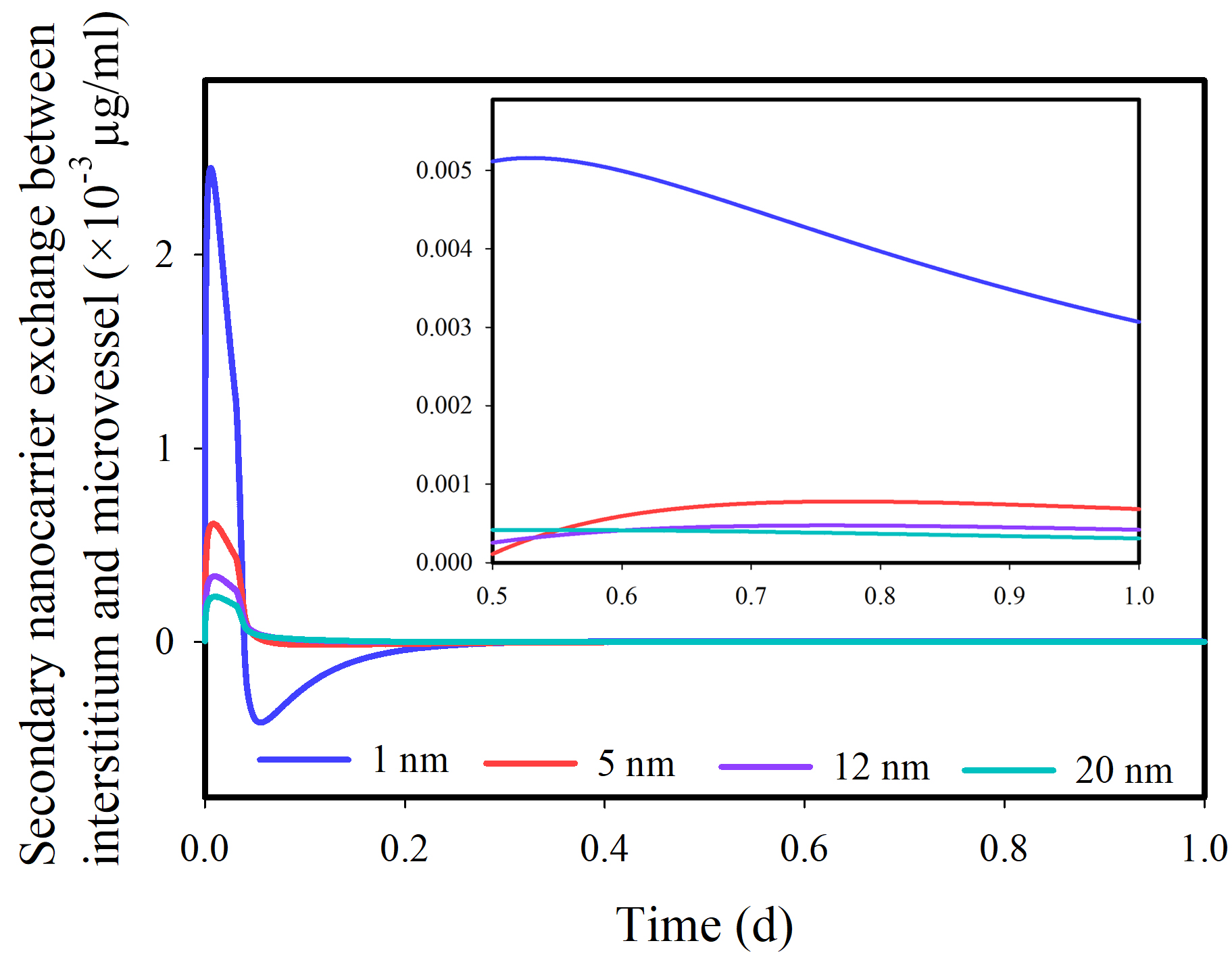 |
| --- |
| Figure S2: Exchange of secondary nanoparticles between microvascular network and extracellular space; 1 nm secondary nanoparticles enter the interstitium at a much higher rate. However, they also leave the interstitium at a higher rate. |

| 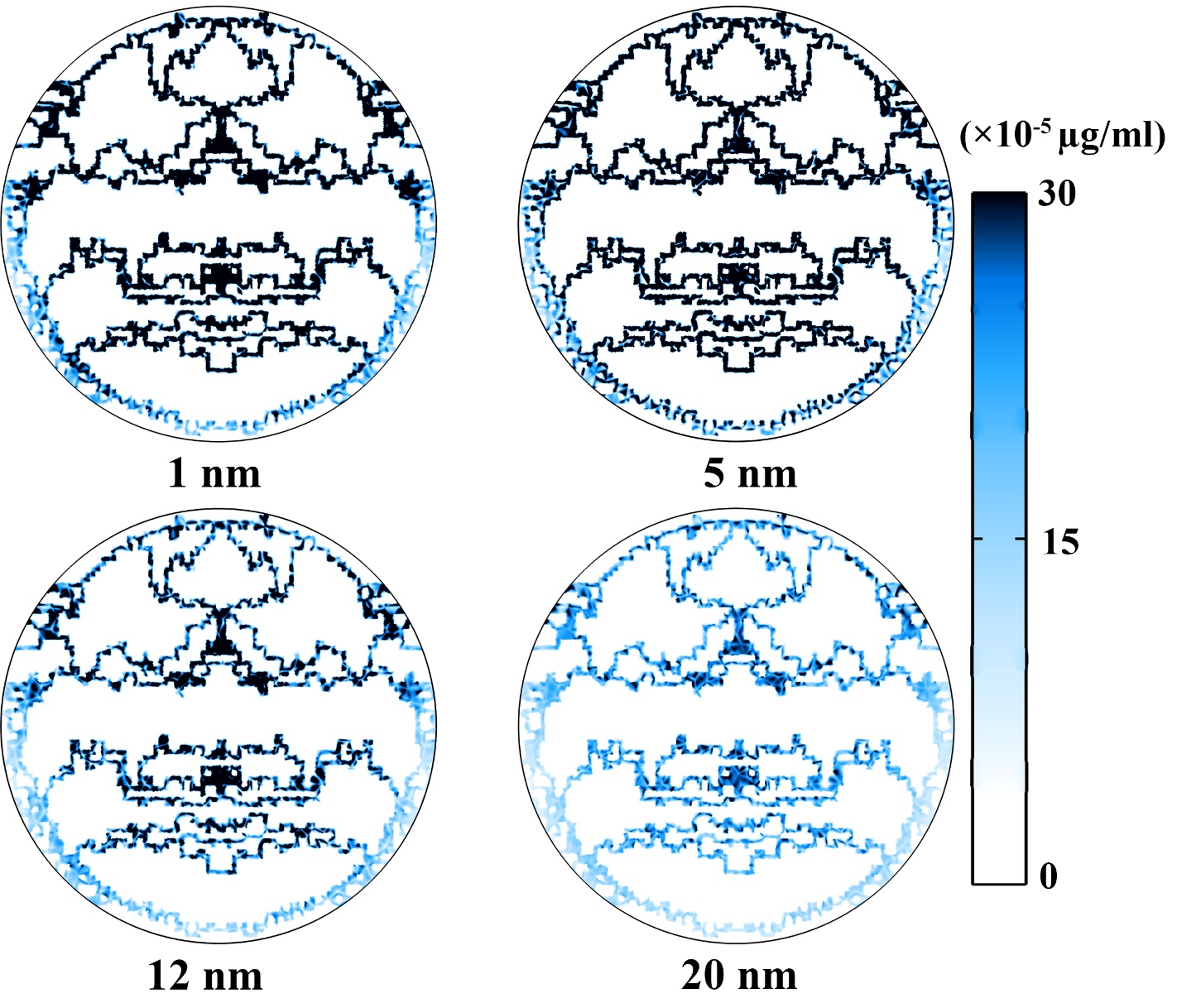 |
| --- |
| Figure S3: Distribution of free drug in the microvascular network; The free drug enters the microvascular which are near the microvascular and eventually enters the systemic circulation. Due to the higher concentration of the drug released from the 1nm nanoparticles, a higher concentration of the drug also enters the microvascular network. |

| 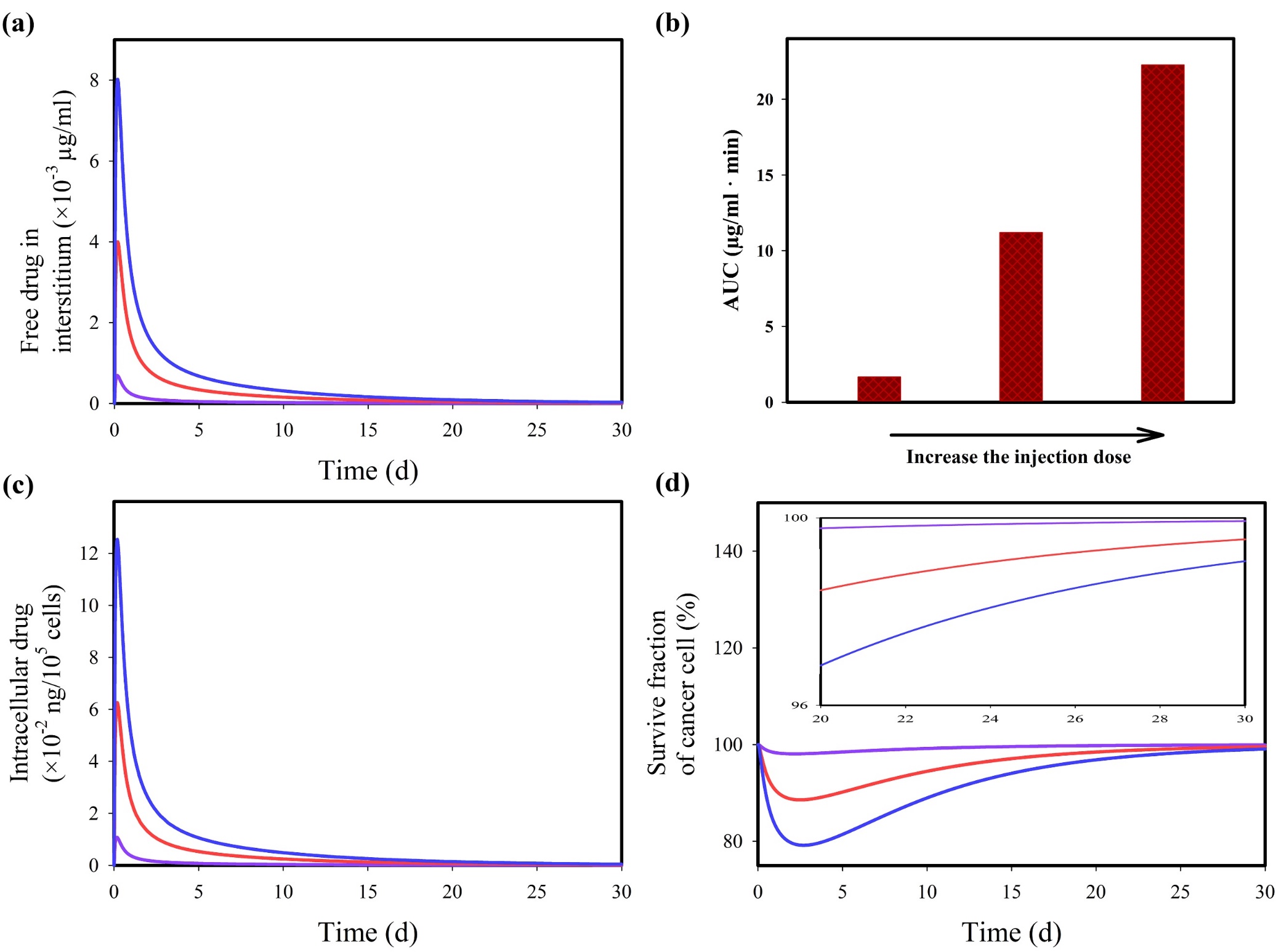 |
| --- |
| Figure S4: Increasing the injected dose increases the concentration of the drug in the extracellular space for a long time and improves the bioavailability of the drug. This causes the cancer cells to be exposed to a higher concentration of free drugs for a longer time. Therefore, cell death and delayed tumor regrowth increase. |

**References**

[1] A.R. Anderson, M.A.J. Chaplain, Continuous and discrete mathematical models of tumor-induced angiogenesis, Bulletin of mathematical biology, 60 (1998) 857-899.

[2] A.R. Anderson, M.A. Chaplain, S. McDougall, A hybrid discrete-continuum model of tumour induced angiogenesis, Modeling Tumor Vasculature, Springer2012, pp. 105-133.

[3] M. Soltani, P. Chen, Numerical modeling of interstitial fluid flow coupled with blood flow through a remodeled solid tumor microvascular network, PloS one, 8 (2013) e67025.

[4] A. Pries, T. Secomb, P. Gaehtgens, Structural adaptation and stability of microvascular networks: theory and simulations, American Journal of Physiology-Heart and Circulatory Physiology, 275 (1998) H349-H360.

[5] T. Alarcón, H.M. Byrne, P.K. Maini, A cellular automaton model for tumour growth in inhomogeneous environment, Journal of theoretical biology, 225 (2003) 257-274.

[6] H.H. Pennes, Analysis of tissue and arterial blood temperatures in the resting human forearm, Journal of applied physiology, 1 (1948) 93-122.

[7] W. Zhan, Mathematical modelling of drug delivery to solid tumour, (2014).

[8] M. Rezaeian, A. Sedaghatkish, M. Soltani, Numerical modeling of high-intensity focused ultrasound-mediated intraperitoneal delivery of thermosensitive liposomal doxorubicin for cancer chemotherapy, Drug delivery, 26 (2019) 898-917.

[9] M. Bailey, V. Khokhlova, O. Sapozhnikov, S. Kargl, L. Crum, Physical mechanisms of the therapeutic effect of ultrasound (a review), Acoustical Physics, 49 (2003) 369-388.

[10] P. Namakshenas, A. Mojra, Numerical study of non-Fourier thermal ablation of benign thyroid tumor by focused ultrasound (FU), Biocybernetics and Biomedical Engineering, 39 (2019) 571-585.

[11] T. Leslie, R. Ritchie, R. Illing, G. Ter Haar, R. Phillips, M. Middleton, B. Bch, F. Wu, D. Cranston, High-intensity focused ultrasound treatment of liver tumours: post-treatment MRI correlates well with intra-operative estimates of treatment volume, The British journal of radiology, 85 (2012) 1363-1370.

[12] S. Crouzet, J.Y. Chapelon, O. Rouviere, F. Mege-Lechevallier, M. Colombel, H. Tonoli-Catez, X. Martin, A. Gelet, Whole-gland ablation of localized prostate cancer with high-intensity focused ultrasound: oncologic outcomes and morbidity in 1002 patients, European urology, 65 (2014) 907-914.

[13] X.-L. Ren, X.-D. Zhou, J. Zhang, G.-B. He, Z.-H. Han, M.-J. Zheng, L. Li, M. Yu, L. Wang, Extracorporeal ablation of uterine fibroids with high‐intensity focused ultrasound: imaging and histopathologic evaluation, Journal of ultrasound in medicine, 26 (2007) 201-212.

[14] T. Huttunen, M. Malinen, J.P. Kaipio, P.J. White, K. Hynynen, A full-wave Helmholtz model for continuous-wave ultrasound transmission, IEEE transactions on ultrasonics, ferroelectrics, and frequency control, 52 (2005) 397-409.

[15] W.L. Nyborg, Sonically produced heat in a fluid with bulk viscosity and shear viscosity, The Journal of the Acoustical Society of America, 80 (1986) 1133-1139.

[16] Y. Huang, B. Gu, C. Liu, J. Stebbing, W. Gedroyc, M. Thanou, X.Y. Xu, Thermosensitive Liposome-Mediated Drug Delivery in Chemotherapy: Mathematical Modelling for Spatio–temporal Drug Distribution and Model-Based Optimisation, Pharmaceutics, 11 (2019) 637.

[17] A.W. El-Kareh, T.W. Secomb, A mathematical model for comparison of bolus injection, continuous infusion, and liposomal delivery of doxorubicin to tumor cells, Neoplasia (New York, NY), 2 (2000) 325.

[18] S. Eikenberry, A tumor cord model for doxorubicin delivery and dose optimization in solid tumors, Theoretical Biology and Medical Modelling, 6 (2009) 16.

[19] R.E. Eliaz, S. Nir, C. Marty, F.C. Szoka, Determination and modeling of kinetics of cancer cell killing by doxorubicin and doxorubicin encapsulated in targeted liposomes, Cancer research, 64 (2004) 711-718.

[20] D.J. Schutt, D. Haemmerich, Effects of variation in perfusion rates and of perfusion models in computational models of radio frequency tumor ablation, Medical physics, 35 (2008) 3462-3470.

[21] F. Henriques Jr, A. Moritz, Studies of thermal injury: I. The conduction of heat to and through skin and the temperatures attained therein. A theoretical and an experimental investigation, The American journal of pathology, 23 (1947) 530.

[22] A. Bhowmik, R. Repaka, S.C. Mishra, K. Mitra, Thermal assessment of ablation limit of subsurface tumor during focused ultrasound and laser heating, Journal of Thermal Science and Engineering Applications, 8 (2016).

[23] T. Balasubramaniam, H. Bowman, Thermal conductivity and thermal diffusivity of biomaterials: A simultaneous measurement technique, (1977).

[24] M. Solovchuk, T.W. Sheu, M. Thiriet, Simulation of nonlinear Westervelt equation for the investigation of acoustic streaming and nonlinear propagation effects, The Journal of the Acoustical Society of America, 134 (2013) 3931-3942.

[25] F. Yuan, M. Dellian, D. Fukumura, M. Leunig, D.A. Berk, V.P. Torchilin, R.K. Jain, Vascular permeability in a human tumor xenograft: molecular size dependence and cutoff size, Cancer research, 55 (1995) 3752-3756.

[26] L.E. Gerlowski, R.K. Jain, Microvascular permeability of normal and neoplastic tissues, Microvascular research, 31 (1986) 288-305.

[27] M.H. Gaber, N.Z. Wu, K. Hong, S.K. Huang, M.W. Dewhirst, D. Papahadjopoulos, Thermosensitive liposomes: extravasation and release of contents in tumor microvascular networks, International Journal of Radiation Oncology• Biology• Physics, 36 (1996) 1177-1187.

[28] J.H. Keenan, F.G. Keyes, Thermodynamic properties of steam, (1936).

[29] D.J. Kerr, A.M. Kerr, R.I. Freshney, S.B. Kaye, Comparative intracellular uptake of adriamycin and 4'-deoxydoxorubicin by nonsmall cell lung tumor cells in culture and its relationship to cell survival, Biochemical pharmacology, 35 (1986) 2817-2823.

[30] S. NAGAOKA, S. KAWASAKI, K. SASAKI, T. NAKANISHI, Intracellular uptake, retention and cytotoxic effect of adriamycin combined with hyperthermia in vitro, Japanese Journal of Cancer Research GANN, 77 (1986) 205-211.

[31] M. Souri, M. Soltani, F. Moradi Kashkooli, Computational modeling of thermal combination therapies by magneto-ultrasonic heating to enhance drug delivery to solid tumors, Scientific reports, 11 (2021) 1-12.

[32] M. Soltani, M. Souri, F. Moradi Kashkooli, Effects of hypoxia and nanocarrier size on pH-responsive nano-delivery system to solid tumors, Scientific Reports, 11 (2021) 1-12.

[33] A. Stéphanou, S.R. McDougall, A.R. Anderson, M.A. Chaplain, Mathematical modelling of the influence of blood rheological properties upon adaptative tumour-induced angiogenesis, Mathematical and Computer Modelling, 44 (2006) 96-123.

[34] F.M. Kashkooli, M. Soltani, M.M. Momeni, A. Rahmim, Enhanced drug delivery to solid tumors via drug-loaded nanocarriers: An image-based computational framework, Frontiers in Oncology, 11 (2021).

[35] F.M. Kashkooli, M. Soltani, M. Rezaeian, C. Meaney, M.-H. Hamedi, M. Kohandel, Effect of vascular normalization on drug delivery to different stages of tumor progression: In-silico analysis, Journal of Drug Delivery Science and Technology, 60 (2020) 101989.

[36] Y. Cai, J. Wu, Z. Li, Q. Long, Mathematical modelling of a brain tumour initiation and early development: a coupled model of glioblastoma growth, pre-existing vessel co-option, angiogenesis and blood perfusion, PloS one, 11 (2016) e0150296.

[37] Y. Cai, J. Zhang, Z. Li, Multi-scale mathematical modelling of tumour growth and microenvironments in anti-angiogenic therapy, Biomedical engineering online, 15 (2016) 685-700.

[38] G. Zhao, J. Wu, S. Xu, M. Collins, Q. Long, C.S. König, Y. Jiang, J. Wang, A. Padhani, Numerical simulation of blood flow and interstitial fluid pressure in solid tumor microcirculation based on tumor-induced angiogenesis, Acta Mechanica Sinica, 23 (2007) 477-483.

[39] G.L. Pishko, G.W. Astary, T.H. Mareci, M. Sarntinoranont, Sensitivity analysis of an image-based solid tumor computational model with heterogeneous vasculature and porosity, Annals of biomedical engineering, 39 (2011) 2360-2373.

[40] A.R. Pries, T.W. Secomb, Microvascular blood viscosity in vivo and the endothelial surface layer, American Journal of Physiology-Heart and Circulatory Physiology, 289 (2005) H2657-H2664.

[41] T.W. Sheu, M.A. Solovchuk, A.W. Chen, M. Thiriet, On an acoustics–thermal–fluid coupling model for the prediction of temperature elevation in liver tumor, International Journal of Heat and Mass Transfer, 54 (2011) 4117-4126.

[42] P. Vaupel, F. Kallinowski, P. Okunieff, Blood flow, oxygen and nutrient supply, and metabolic microenvironment of human tumors: a review, Cancer research, 49 (1989) 6449-6465.

[43] A. Gasselhuber, M.R. Dreher, A. Partanen, P.S. Yarmolenko, D. Woods, B.J. Wood, D. Haemmerich, Targeted drug delivery by high intensity focused ultrasound mediated hyperthermia combined with temperature-sensitive liposomes: computational modelling and preliminary in vivo validation, International Journal of Hyperthermia, 28 (2012) 337-348.

[44] P.S. Tofts, G. Brix, D.L. Buckley, J.L. Evelhoch, E. Henderson, M.V. Knopp, H.B. Larsson, T.Y. Lee, N.A. Mayr, G.J. Parker, Estimating kinetic parameters from dynamic contrast‐enhanced T1‐weighted MRI of a diffusable tracer: standardized quantities and symbols, Journal of Magnetic Resonance Imaging: An Official Journal of the International Society for Magnetic Resonance in Medicine, 10 (1999) 223-232.

[45] D.M. Brizel, B. Klitzman, J.M. Cook, J. Edwards, G. Rosner, M.W. Dewhirst, A comparison of tumor and normal tissue microvascular hematocrits and red cell fluxes in a rat window chamber model, International Journal of Radiation Oncology* Biology* Physics, 25 (1993) 269-276.

[46] F. Yuan, M. Leunig, D.A. Berk, R.K. Jain, Microvascular permeability of albumin, vascular surface area, and vascular volume measured in human adenocarcinoma LS174T using dorsal chamber in SCID mice, Microvascular research, 45 (1993) 269-289.

[47] W. Zhan, W. Gedroyc, X.Y. Xu, Towards a multiphysics modelling framework for thermosensitive liposomal drug delivery to solid tumour combined with focused ultrasound hyperthermia, Biophysics Reports, 5 (2019) 43-59.

[48] Y.-M.F. Goh, H.L. Kong, C.-H. Wang, Simulation of the delivery of doxorubicin to hepatoma, Pharmaceutical Research, 18 (2001) 761-770.

[49] N.Z. Wu, B. Klitzman, G. Rosner, D. Needham, M.W. Dewhirst, Measurement of material extravasation in microvascular networks using fluorescence video-microscopy, Microvascular research, 46 (1993) 231-253.

[50] R.K. Jain, Transport of molecules in the tumor interstitium: a review, Cancer research, 47 (1987) 3039-3051.

[51] E.A. Swabb, J. Wei, P.M. Gullino, Diffusion and convection in normal and neoplastic tissues, Cancer research, 34 (1974) 2814-2822.

[52] L.J. Nugent, R.K. Jain, Extravascular diffusion in normal and neoplastic tissues, Cancer research, 44 (1984) 238-244.

[53] M.B. Wolf, P.D. Watson, D. Scott 2nd, Integral-mass balance method for determination of solvent drag reflection coefficient, American Journal of Physiology-Heart and Circulatory Physiology, 253 (1987) H194-H204.

[54] C. Liu, J. Krishnan, X.Y. Xu, Investigating the effects of ABC transporter-based acquired drug resistance mechanisms at the cellular and tissue scale, Integrative Biology, 5 (2013) 555-568.

[55] J. Robert, A. Illiadis, B. Hoerni, J.-P. Cano, M. Durand, C. Lagarde, Pharmacokinetics of adriamycin in patients with breast cancer: correlation between pharmacokinetic parameters and clinical short-term response, European Journal of Cancer and Clinical Oncology, 18 (1982) 739-745.

[56] L.Z. Benet, P. Zia-Amirhosseini, Basic principles of pharmacokinetics, Toxicologic pathology, 23 (1995) 115-123.

[57] K.A. Rodvold, D.A. Rushing, D.A. Tewksbury, Doxorubicin clearance in the obese, Journal of Clinical Oncology, 6 (1988) 1321-1327.

[58] A. Gasselhuber, M.R. Dreher, A. Negussie, B.J. Wood, F. Rattay, D. Haemmerich, Mathematical spatio-temporal model of drug delivery from low temperature sensitive liposomes during radiofrequency tumour ablation, International Journal of Hyperthermia, 26 (2010) 499-513.

[59] T. Stylianopoulos, E.-A. Economides, J.W. Baish, D. Fukumura, R.K. Jain, Towards optimal design of cancer nanomedicines: Multi-stage nanoparticles for the treatment of solid tumors, Annals of biomedical engineering, 43 (2015) 2291-2300.

[60] K. Igarashi, H. Cabral, T. Hong, Y. Anraku, F. Mpekris, T. Stylianopoulos, T. Khan, A. Matsumoto, K. Kataoka, Y. Matsumoto, Vascular Bursts Act as a Versatile Tumor Vessel Permeation Route for Blood‐Borne Particles and Cells, Small, 17 (2021) 2103751.

[61] H. Cabral, Y. Matsumoto, K. Mizuno, Q. Chen, M. Murakami, M. Kimura, Y. Terada, M. Kano, K. Miyazono, M. Uesaka, Accumulation of sub-100 nm polymeric micelles in poorly permeable tumours depends on size, Nature nanotechnology, 6 (2011) 815-823.

[62] Y. Anraku, A. Kishimura, A. Kobayashi, M. Oba, K. Kataoka, Size-controlled long-circulating PICsome as a ruler to measure critical cut-off disposition size into normal and tumor tissues, Chemical Communications, 47 (2011) 6054-6056.

[63] V.P. Chauhan, T. Stylianopoulos, J.D. Martin, Z. Popović, O. Chen, W.S. Kamoun, M.G. Bawendi, D. Fukumura, R.K. Jain, Normalization of tumour blood vessels improves the delivery of nanomedicines in a size-dependent manner, Nature nanotechnology, 7 (2012) 383-388.

[64] A. Gabizon, R. Catane, B. Uziely, B. Kaufman, T. Safra, R. Cohen, F. Martin, A. Huang, Y. Barenholz, Prolonged circulation time and enhanced accumulation in malignant exudates of doxorubicin encapsulated in polyethylene-glycol coated liposomes, Cancer research, 54 (1994) 987-992.
