# Supplementary figures and images for: Stimuli-sensitive nano-drug delivery with programmable size changes to enhance accumulation of therapeutic agents in tumors
Source: Drug Deliv. 2023 Mar 9;30(1):2186312. doi: 10.1080/10717544.2023.2186312 (PMC10013474; doi:10.1080/10717544.2023.2186312)

## Slide 1
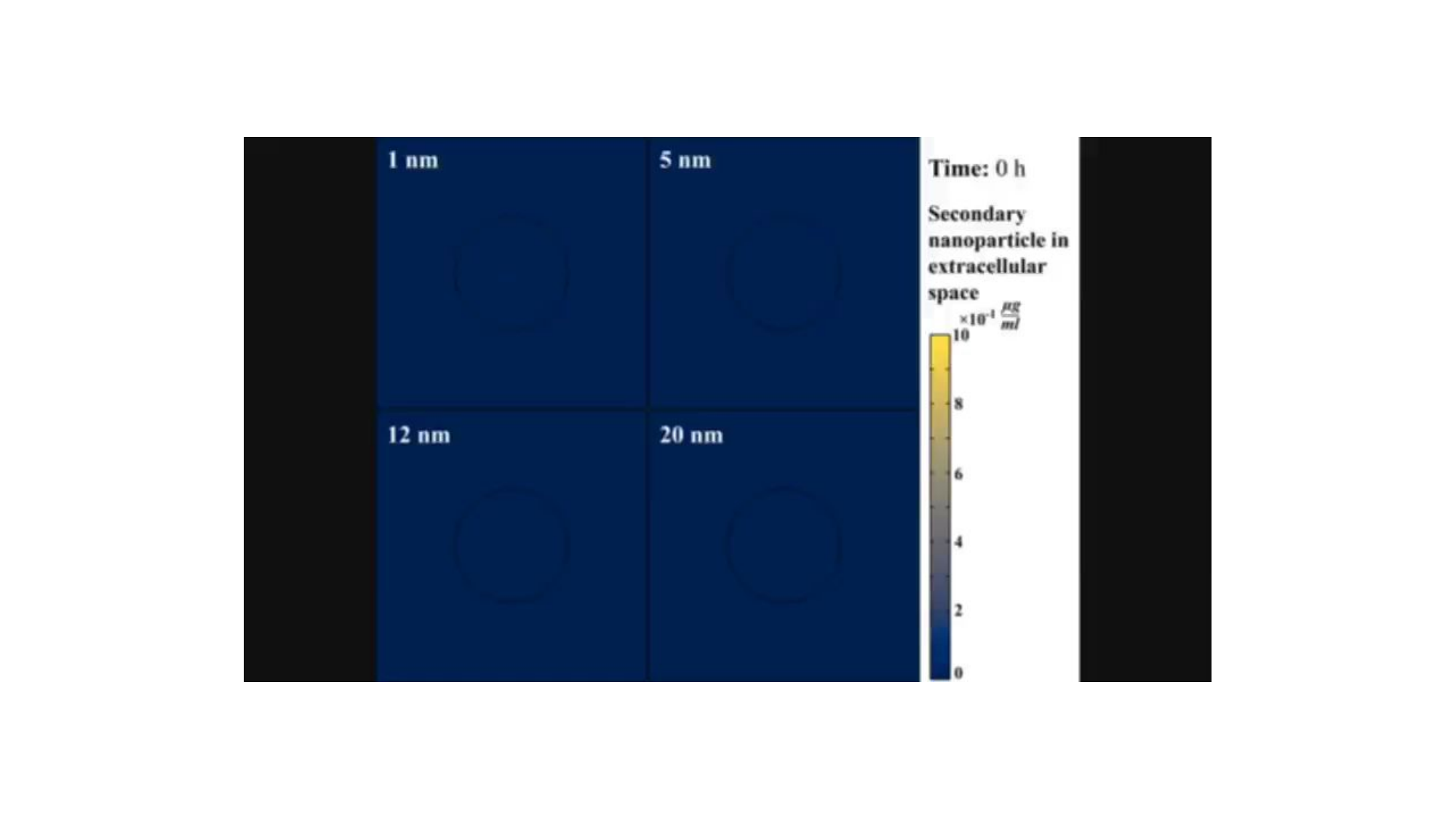

Supplement: Supplemental Material [file IDRD_A_2186312_SM8290.zip › Supplementary Video and File/Supplementary Video 1 .pptx]

## Slide 1
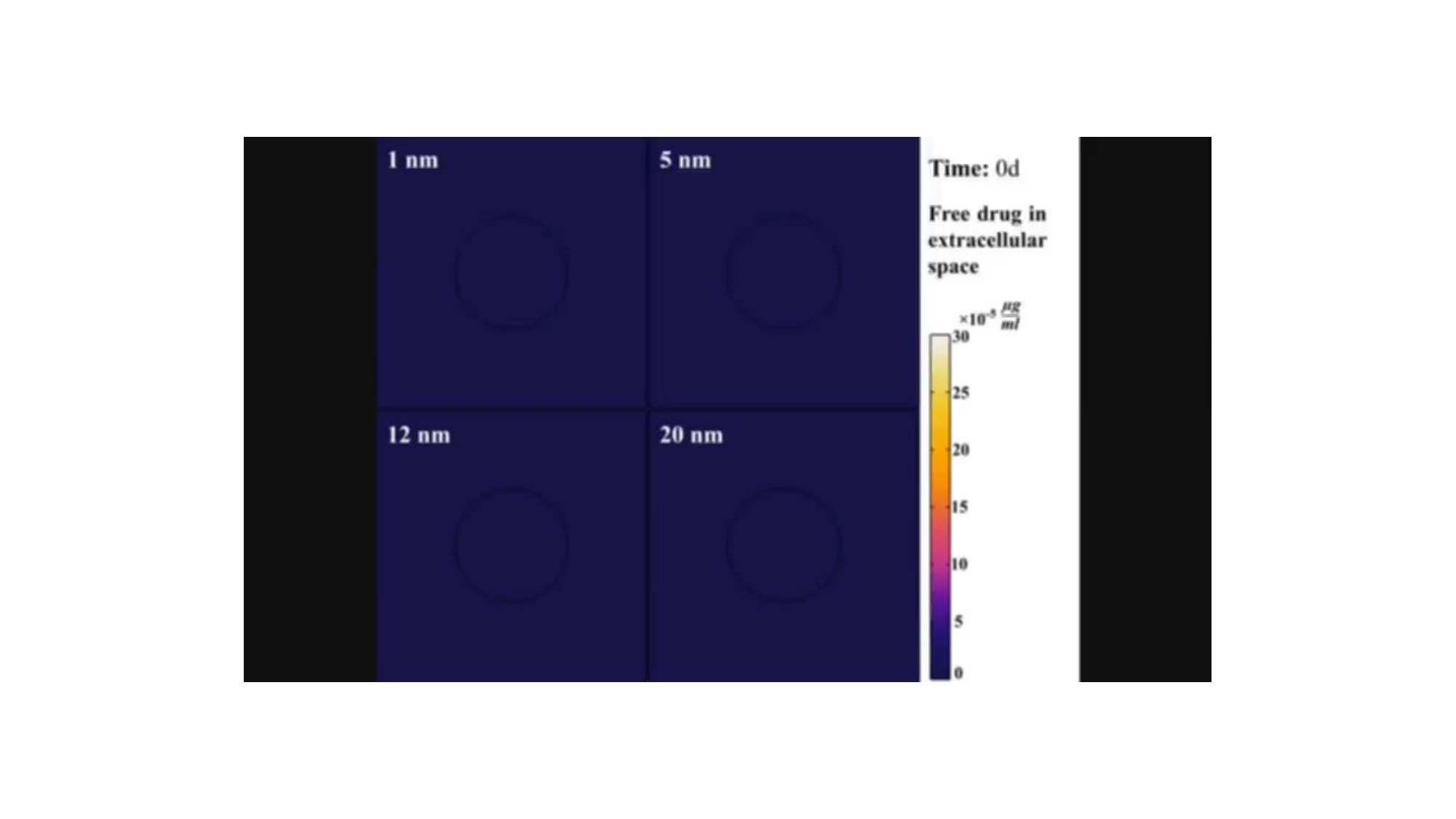

Supplement: Supplemental Material [file IDRD_A_2186312_SM8290.zip › Supplementary Video and File/Supplementary Video 2.pptx]
